# Functional Heterogeneity of Cell Populations Increases Robustness of Pacemaker Function in a Numerical Model of the Sinoatrial Node Tissue

**DOI:** 10.3389/fphys.2022.845634

**Published:** 2022-04-27

**Authors:** Alexander V. Maltsev, Michael D. Stern, Edward G. Lakatta, Victor A. Maltsev

**Affiliations:** Intramural Research Program, National Institute on Aging, NIH, Baltimore, MD, United States

**Keywords:** sinoatrial node, coupled-clock pacemaker system, numerical modeling, calcium release, sarcoplasmic reticulum, cardiac arrhythmia, sinus node arrest, sick sinus syndrome

## Abstract

Each heartbeat is initiated by specialized pacemaker cells operating within the sinoatrial node (SAN). While individual cells within SAN tissue exhibit substantial heterogeneity of their electrophysiological parameters and Ca cycling, the role of this heterogeneity for cardiac pacemaker function remains mainly unknown. Here we investigated the problem numerically in a 25 × 25 square grid of connected coupled-clock Maltsev-Lakatta cell models. The tissue models were populated by cells with different degree of heterogeneity of the two key model parameters, maximum L-type Ca current conductance (*g*_*CaL*_) and sarcoplasmic reticulum Ca pumping rate (*P*_*up*_). Our simulations showed that in the areas of *P*_*up*_-*g*_*CaL*_ parametric space at the edge of the system stability, where action potential (AP) firing is absent or dysrhythmic in SAN tissue models populated with identical cells, rhythmic AP firing can be rescued by populating the tissues with heterogeneous cells. This robust SAN function is synergistic with respect to heterogeneity in *g*_*CaL*_ and *P*_*up*_ and can be further strengthened by clustering of cells with similar properties. The effect of cell heterogeneity is not due to a simple summation of activity of intrinsically firing cells naturally present in heterogeneous SAN; rather AP firing cells locally and critically interact with non-firing/dormant cells. When firing cells prevail, they recruit many dormant cells to fire, strongly enhancing overall SAN function; and vice versa, prevailing dormant cells suppress AP firing in cells with intrinsic automaticity and halt SAN function. The transitions between firing and non-firing states of the system are sharp, resembling phase transitions in statistical physics. Furthermore, robust function of heterogeneous SAN tissue requires weak cell coupling, a known property of the central area of SAN where cardiac impulse emerges; stronger cell coupling reduces AP firing rate and ultimately halts SAN automaticity at the edge of stability.

## Highlights

**Non-firing zone**—the area of *P*_*up*_–*g*_*CaL*_ parametric space where individual SAN cells lack automaticity
**Chaotic firing zone**—the area of *P*_*up*_–*g*_*CaL*_ parametric space where individual SAN cells generate chaotic (dysrhythmic) AP firing
**Rhythmic firing zone**—the area of *P*_*up*_–*g*_*CaL*_ parametric space where individual SAN cells generate rhythmic AP firing
**The bifurcation line**—the border line in *P*_*up*_–*g*_*CaL*_ parametric space separating firing, non-firing, and chaotic firing zones
**Robustness**—ability of SAN tissue to generate rhythmic APs in a wide range of model parameters: the larger rhythmic firing zone, the higher robustness of the tissue model
**The coupled-clock system**: a coupled system of ion current oscillator in the cell membrane and intracellular Ca oscillator of the SR inside a SAN cell. According to the coupled-clock theory, the oscillators critically interact via multiple Ca- and voltage-dependent mechanisms to generate normal SAN cell automaticity.

## Introduction

The sinoatrial node (SAN) of the right atrium is the heart’s primary pacemaker, providing both robust and flexible automatic electrical depolarizations that capture the surrounding atrial myocardium and drive the heartbeat. These electrical depolarizations are caused by concurrent operation of pacemaker cells residing in SAN tissue. These cells are electrical oscillators that spontaneously generate rhythmic changes of their membrane potential (*V*_*m*_), producing relatively periodic spontaneous action potentials (APs) (reviews (Mangoni and Nargeot, 2008; Maltsev et al., 2014)). The SAN was discovered in 1907 (Keith and Flack, 1907). However, despite enormous amount of experimental data, numerical modeling, and plausible theories, the SAN operation remains still mysterious after all these years (Weiss and Qu, 2020).

The enigma of SAN function stems from the fact that the SAN is an extremely complex heterogeneous tissue regarding individual cell properties and their connections within the tissue (Boyett et al., 2000). Furthermore, at the cellular level, individual SAN cells operate via a complex coupled-clock paradigm (Maltsev and Lakatta, 2009; Lakatta et al., 2010), i.e. they possess not only an electrical oscillator within their cell surface membranes, but also an intracellular oscillator, sarcoplasmic reticulum (SR) that cycles Ca in-and-out via SR Ca pump (SERCA2a) and release channels, ryanodine receptors (RyRs). Ca releases via RyRs interact with Na/Ca exchanger and L-type Ca current (*I*_*CaL*_), and together they drive diastolic depolarization via a positive feedback mechanism (dubbed ignition (Lyashkov et al., 2018)). The synchronization of molecular actions, in turn, is driven by enhanced enzymatic activity of Ca-activated adenylate cyclases (AC1 and AC8) (Mattick et al., 2007; Younes et al., 2008) and protein kinases (PKA and CaMKII) (Vinogradova and Lakatta, 2009; Lakatta et al., 2010) that modulate respective ion channel properties and accelerate Ca pumping.

**FIGURE 1:**
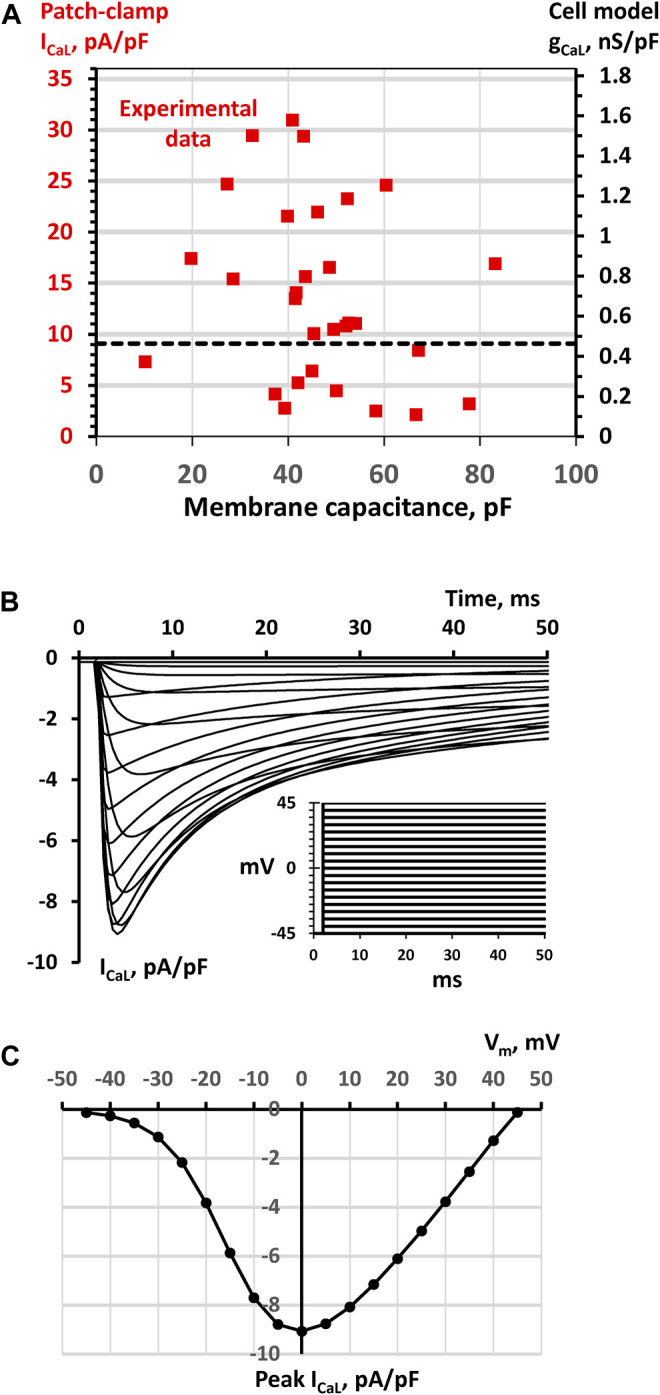
SAN cell model simulations to match model parameter *g*_*CaL*_ to *I*_*CaL*_ density measured experimentally **(A)** A double Y scale plot of experimental data points of *I*_*CaL*_ densities measured by whole-cell patch clamp in a large population of SAN cells (red squares, left *Y* axis, replotted from (Monfredi et al., 2018)) and respective model *g*_*CaL*_ parameter values (right *Y* axis) **(B)** Traces of *I*_*CaL*_/*V*_*m*_ relationship simulated in Maltsev-Lakatta single cell model with the basal state value of *g*_*CaL*_ = 0.464 nS/pF using voltage clamp protocol (inset) and conditions similar to those in experimental study of (Monfredi et al., 2018). The family of *I*_*CaL*_ traces was simulated by applying a series of square voltage pulses from a holding potential of -45 mV with a step of 5 mV up to 45 mV (inset). Before each stimulation, *I*_*CaL*_ activation and inactivation gating variables *f*_*L*_ and *d*_*L*_ were set to their steady-state values. To closely simulate experimental whole-cell patch clamp conditions in (Monfredi et al., 2018), 5 mM EGTA (equilibrated to 100 nM of free Ca) was added to Ca formulations of cell submembrane space and cytoplasm **(C)** Respective *I*_*CaL*_/*V*_*m*_ relationship calculated for peak *I*_*CaL*_. The *I*_*CaL*_ peak of 9.068 pA/pF at 0 mV was set as a bridge between experimental data and model *g*_*CaL*_ = 0.464 nS/pF (black dash line in panel A).

The expression of Ca cycling proteins, and membrane ion channels vary substantially among individual SAN cells (Honjo et al., 1996; Honjo et al., 1999; Lei et al., 2001; Musa et al., 2002; Monfredi et al., 2017). For example, *I*_*CaL*_ density varies by an order of magnitude (Monfredi et al., 2017) (Figure 1A, red squares). This results in a large degree of functional heterogeneity among individual cells, ranging from frequently and rhythmic AP firing to infrequently and dysrhythmic firing, to nonfiring cells (recently dubbed “dormant” cells) (Kim et al., 2018; Tsutsui et al., 2018; Tsutsui et al., 2021). The SAN cell population is also extremely heterogeneous with respect to the AP firing rate increase in response to β-adrenergic stimulation (Kim et al., 2021).

At the SAN tissue level, it was initially thought that a “master” pacemaker cell or a leading pacemaker center dictates the excitation rate and rhythm of other SAN pacemaker cells (Sano et al., 1978; Bleeker et al., 1980). Subsequent studies suggested that individual cells mutually entrain each other to fire APs with a common period (dubbed “democratic” process) (Jalife, 1984; Michaels et al., 1987). More recent high-resolution imaging of intact SAN tissue at a single cell level, however, demonstrated that while majority of SAN cells indeed fire synchronously with a common period, many cells fire “at will”, i.e. at various rates and irregularly, or remain silent, generating only local Ca releases (Bychkov et al., 2020; Fenske et al., 2020). Thus, how structural and functional heterogeneities within and among SAN cells give rise to synchronized AP firing at the SAN exits remains a mystery at the frontier of pacemaker research (Clancy and Santana, 2020; Weiss and Qu, 2020).

Numerical modeling is a powerful approach to study complex cell interactions. Here we used a modified 2-dimensional model of SAN tissue developed by Campana (Campana, 2015), featuring faster parallel computing via graphics processing unit (GPU) to test the hypothesis that functional heterogeneity of cells within SAN tissue increases robustness of its pacemaker function. To this end, we adapted the model of SAN tissue to include diverse cell populations with respect to maximum SR Ca pumping rate (*P*_*up*_) and *I*_*CaL*_ conductance (*g*_*CaL*_) as observed experimentally (Honjo et al., 1996; Monfredi et al., 2017) (Figure 1A; Table 1) and examined SAN function at the edge of the system stability at different cell-to-cell coupling strength.

**TABLE 1:** Parameter values in each model simulation scenario (column #) and results of our simulations during evaluation time interval from 5 to 7.5 s after simulation onset: the percentage of AP firing cells in tissue, the percentage of cells firing APs in isolation, and AP cycle length average with its standard deviation (SD) calculated for all AP firing cells populating SAN tissue model (25 × 25 square grid) in each scenario. N.D. means “Not Defined” in scenarios, in which SAN tissue did not fire APs. Numerical simulations for the two sets of scenarios (2a, 2b, and 2c) or (5a, 5b, and 5c) were performed with different seed values for random number generator to create different cells populations with respect to *g*_*CaL*_ or *P*_*up*_, respectively. *44.48% of AP firing cells reflects firing only during initial part of the evaluation time interval in scenario 5b; the tissue model ceased firing at about 6 s (see Supplementary Movies S5B).

#	ρ MΩ×m	Random or Cluster	*P*_*up*_, min, mM/s	*P*_*up*_, max, mM/s	*g*_*CaL*_, min, nS/pF	*g*_*CaL*_, max, nS/pF	% firing cells, Separate	% firing cells, Tissue	Cycle length mean, ms	Cycle length SD, ms
1	10^4^	random	7	7	0.14	0.54	46	64.64	407.17	5.81
2a	10^4^	random	5	5	0.17	0.57	46	70.4	430.29	5.34
2b	10^4^	random	5	5	0.17	0.57	46	71.68	433.91	23.21
2c	10^4^	random	5	5	0.17	0.57	46	73.44	434.31	52.63
3	10^4^	random	3	3	0.21	0.61	47.5	83.2	458.62	90.49
4	10^4^	random	2	12	0.34	0.34	39	0	N.D.	N.D.
5a	10^4^	random	0	10	0.37	0.37	40	97.92	481.86	115.85
5b	10^4^	random	0	10	0.37	0.37	40	44.48*	514.44	56.21
5c	10^4^	random	0	10	0.37	0.37	40	99.84	518.74	152.37
6	10^4^	random	0	6	0.41	0.41	40	100	506.91	6.27
7	10^4^	random	7	7	0.3	0.38	30	0	N.D.	N.D.
8	10^4^	random	2	12	0.3	0.38	42	69.12	447.29	98.18
9	10^4^	*g*_*CaL*_ cluster	7	7	0.3	0.38	30	60.8	457.99	2.16
10	10^4^	*P*_*up*_ cluster	2	12	0.34	0.34	39	66.4	479.43	277.62
11	10^3^	random	5	5	0.17	0.57	46	97.12	454.41	2.34
12	10^3^	random	0	10	0.37	0.37	40	100	759.24	68.5
13	1	random	5	5	0.17	0.57	46	0	N.D.	N.D.
14	1	random	0	10	0.37	0.37	40	0	N.D.	N.D.
15	10^4^	random	5	5	0.24	0.64	63	98.56	413.41	2.67
16	10^4^	random	5	15	0.37	0.37	88	100	364.42	3.28
17	10^4^	random	7	7	0.34	0.42	77.5	100	431.68	1.74
18	10^4^	random	7	17	0.34	0.34	88	100	353.23	3.1

## Materials and Methods

### Single Cell Model

Our SAN tissue model is comprised of single cell models developed by Maltsev and Lakatta in 2009 (Maltsev and Lakatta, 2009). Each model encompasses a system of 29 first order differential equations (see state variables *y*_*1*_ *-y*_*29*_ in Table 2). This was the first SAN cell numerical model featuring coupled operation of Ca and membrane clocks. The model predicted the contribution of spontaneous Ca release during diastolic depolarization via activation of inward Na/Ca exchanger current that explained numerous experimental data. The computer code for the original model can be freely downloaded in CellML format (maltsev_2009_paper.cellml) at http://models.cellml.org/workspace/maltsev_2009 and executed using the Cellular Open Resource (COR) software developed at the University of Oxford by Garny et al. (Garny et al., 2009) (for recent development of the COR see http://www.opencor.ws/).

**TABLE 2:** State variables *y*_*1*_ *-y*_*29*_ with their descriptions and initial values assigned to all cells in our SAN tissue model simulations.

#	Variable	Description	Initial Value
Ca cycling			
*y*_*1*_	*Ca*_*i*_	[Ca] in myoplasm, mM	0.0001
*y*_*2*_	*Ca*_*sub*_	[Ca] in submembrane space, mM	0.000223
*y*_*3*_	*Ca*_*jSR*_	[Ca] in the junctional SR (jSR), mM	0.029
*y*_*4*_	*Ca*_*nSR*_	[Ca] in the network SR (nSR), mM	1.35
*y*_*5*_	*f*_*TC*_	Fractional occupancy of the troponin-Ca site by Ca in myoplasm	0.02
*y*_*6*_	*f*_*TMC*_	Fractional occupancy of the troponin-Mg site by Ca in myoplasm	0.22
*y*_*7*_	*f*_*TMM*_	Fractional occupancy of the troponin-Mg site by Mg in myoplasm	0.69
*y*_*8*_	*f*_*CMi*_	Fractional occupancy of calmodulin by Ca in myoplasm	0.042
*y*_*9*_	*f*_*CMs*_	Fractional occupancy of calmodulin by Ca in submembrane space	0.089
*y*_*10*_	*f*_*CQ*_	Fractional occupancy of calsequestrin by Ca in junctional SR	0.032
*y*_*11*_	*R*	RyR reactivated (closed) state	0.75
*y*_*12*_	*O*	RyR open state	3.4 · 10^–6^
*y*_*13*_	*I*	RyR inactivated state	1.1 · 10^–6^
*y*_*14*_	*RI*	RyR RI state	0.25
Electrophysiology			
*y*_*15*_	*V*_*m*_	Membrane potential, mV	-65
*y*_*16*_	*d*_*L*_	*I*_CaL_ activation	0
*y*_*17*_	*f*_*L*_	*I*_CaL_ voltage-dependent inactivation	1
*y*_*18*_	*f*_*Ca*_	*I*_CaL_ Ca dependent inactivation	1
*y*_*19*_	*p*_*aF*_	*I*_Kr_ fast activation	0
*y*_*20*_	*p*_*aS*_	*I*_Kr_ slow activation	0
*y*_*21*_	*p*_*i*_	*I*_Kr_ inactivation	1
*y*_*22*_	*n*	*I*_Ks_ activation	0
*y*_*23*_	*y*	*I*_f_ activation	1
*y*_*24*_	*d*_*T*_	*I*_CaT_ activation	0
*y*_*25*_	*f*_*T*_	*I*_CaT_ inactivation	1
*y*_*26*_	*q*	*I*_to_ inactivation	1
*y*_*27*_	*r*	*I*_to_ and *I*_sus_ activation	0
*y*_*28*_	*q*_*a*_	*I*_st_ activation	0
*y*_*29*_	*q*_*i*_	*I*_st_ inactivation	1

Since 2009, the model has been tested, modified and used in numerous applications (Maltsev and Lakatta, 2010; Kurata et al., 2012; Severi et al., 2012; Maltsev and Lakatta, 2013; Campana, 2015; Sirenko et al., 2016; Kim et al., 2018; Lyashkov et al., 2018; Yang et al., 2021). According to the coupled-clock theory, a coupled system of Ca and surface membrane “clocks” can provide the robustness and flexibility required for normal pacemaker cell function. A simple numerical release-pumping-delay Ca oscillator is capable of generating any frequency between 1.3 and 6.1 Hz, but cannot generate high-amplitude signals without the assistance of membrane clocks (Maltsev and Lakatta, 2009). The coupled-clock system utilizes the greater flexibility of the SR Ca clock while simultaneously accounting for the large Ca oscillation amplitudes fueled via sarcolemmal Ca influx associated with *I*_*CaL*_. A modern interpretation of the coupled-clock function is that Ca clock operates via a criticality mechanism, whereas the membrane clock operates as a limited cycle oscillator, comprising a highly efficient, synergistic pacemaker cell system (Weiss and Qu, 2020).

### Multi-Cellular Model of SAN

The present study examined performance of SAN tissue that was modelled as a square grid of 25 by 25 of Maltsev-Lakatta cell models (Maltsev and Lakatta, 2009), with each cell being connected to its four neighbors (except those on the border). Our model was an adapted version of the tissue model originally developed by Campana (Campana, 2015). The final numerical approximation of reaction-diffusion equation to compute voltage in a 2-dimensional network of SAN cells was as follows (Equation (2.7) in (Campana, 2015)):

$$V_{m}\left[m\right]=x\left[n\right]+step\times \left(rad^{2}\frac{\pi }{\rho C_{m}deltx}V_{net}-\frac{I_{ion}\left[n\right]+stim}{C_{m}}\right)$$

**FIGURE 2:**
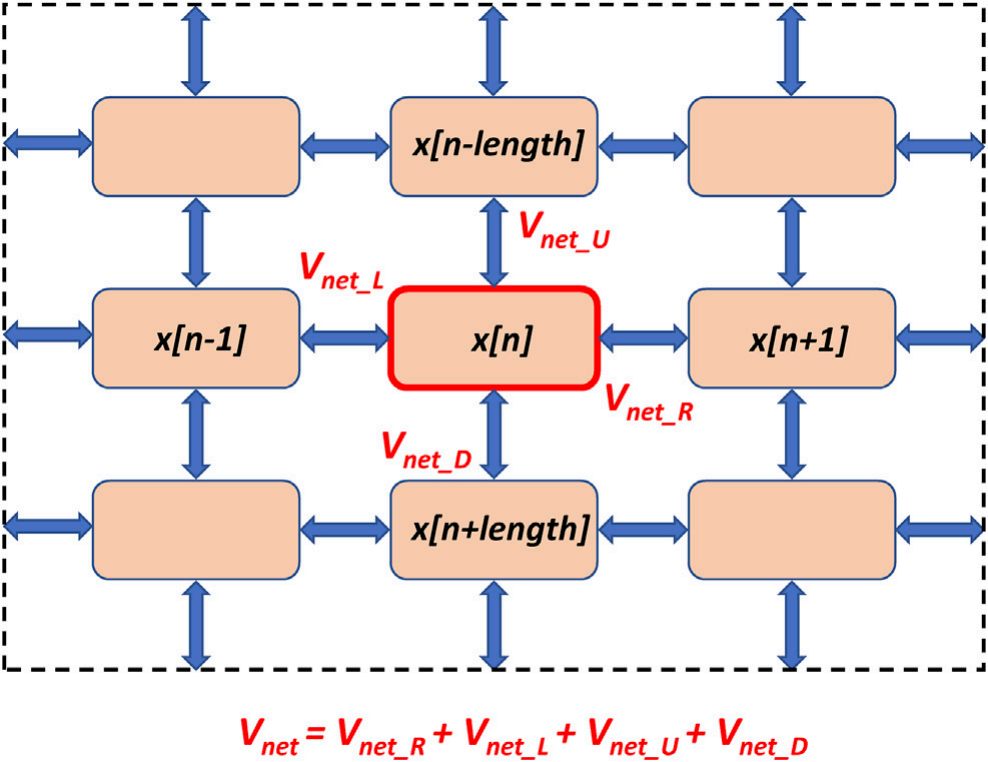
A schematic illustration of SAN tissue structure and calculation of the network voltage (*V*_*net*_) for a cell with index *n* in Campana model (Campana, 2015). Pink squares denote individual SAN cells (Maltsev-Lakatta models) located at the nodes of a square grid. The cells interact via intercellular conductances placed uniformly at the grid’s edges (shown by blue double-headed arrows). *V*_*net*_ is the sum of voltages from the four neighboring cells (right, left, up, and down). Shown is only a 3 × 3 section the network; the tissue continues beyond the dash lines to form the entire 25 × 25 cell network used in our simulations.

Where *V*_*m*_ *[m]* is *V*_*m*_ of the cell with index *m* within the cellular network represented by a vector (1..number of cells); *x* is the state variable array; for each time step, *m* and *n* denote the same cell, only in two different vectors and *x[n]* represents the value assumed by *V*_*m*_ *[m]* at the previous time step; *V*_*net*_ is the sum of voltages from the four neighboring cells (right, left, up, and down): *V*_*net*_ *= V*_*net_R*_ *+ V*_*net_L*_ *+ V*_*net_U*_ *+ V*_*net_D*_ (Figure 2); *step* is the model integration time step of 0.005 ms; *rad* is the cell radius of 4 μm; *ρ* is the intracellular resistivity of 10^4^ MΩ·m; *C*_*m*_ is the electrical membrane capacitance of 32 pF; *deltx* is the cell length of 70 μm; *I*_*ion*_ is the sum of all ion currents; *stim* is stimulus current (*stim* = 0 in our study).

### Simulation of Functional Heterogeneity in SAN Tissue Models

Tissue heterogeneity was introduced in the model by varying *P*_*up*_ and *g*_*CaL*_, the two key parameters of the coupled-clock system (Maltsev and Lakatta, 2009). *P*_*up*_ determines the rate at which Ca is pumped into the SR by SERCA2a to reach a threshold of Ca load required for activation of Ca release, i.e. a central part in the timing mechanism of the coupled-clock system (Maltsev and Lakatta, 2009; Imtiaz et al., 2010; Vinogradova et al., 2010; Stern et al., 2014; Maltsev et al., 2017b). Our cell model approximated Ca uptake flux *j*_up_ to the network SR compartment as a function of cytoplasmic [Ca] (*Ca*_*i*_) as suggested originally by Luo and Rudy (Luo and Rudy, 1994) in ventricular cells and subsequently used in SAN cell models, e.g. (Kurata et al., 2002; Maltsev and Lakatta, 2009; Severi et al., 2012):

$$j_{up}\;=\;P_{up}/\left(1\;+\;K_{\mathrm{up}}/{Ca}_{\text{i}}\right)\;$$

where *K*_up_ = 0.6 · 10^–3^ mM is *Ca*_*i*_ for a half-maximal Ca uptake to the network SR. Some early studies suggested that phospholamban phosphorylation increases the apparent affinity of the Ca-ATPase for Ca (Tada and Inui, 1983), i.e. *K*_up_ in our formulation. But further studies suggested that dephosphorylated phospholamban acts as an inhibitor of the Ca-ATPase and that phosphorylation releases the inhibition (Inui et al., 1986), i.e. *P*_up_ **.** Phospholamban can also operate as an oligomer (Glaves et al., 2019) pointing to a complex regulation of SERCA function. Because our study is not focused on SERCA regulation, we chose only one parameter *P*_up_ representing the performance of the Ca clock in our simulations of the coupled clock system (Maltsev and Lakatta, 2009).

There is an additional reason to examine specifically *P*_up_ in our tissue models: *P*_up_ and *g*_*CaL*_ and their combinations are the most examined parameters in previous theoretical studies with respect to SAN cell function (Maltsev and Lakatta, 2009, 2010; Kurata et al., 2012; Severi et al., 2012; Maltsev and Lakatta, 2013), including 2-dimensional parametric sensitivity analysis, i.e. *P*_up_-*g*_*CaL*_ bifurcation diagram (Maltsev and Lakatta, 2009) that is used in the present study. This approach allowed us to plan and interpret our simulations not only for variations of either *P*_up_ or *g*_*CaL*_, but importantly for simultaneous variations of *P*_up_ and *g*_*CaL*_, i.e. interactions of the clocks, providing new insights into the system function.

*I*_*CaL*_ as a function of *V*_*m*_, submembrane [Ca] (*Ca*_*sub*_), and time (*t*) was approximated as follows (for more details, see (Maltsev and Lakatta, 2009)):

$$I_{\mathrm{CaL}}{=C}_{m}{\mathrm{\cdot g}}_{\mathrm{CaL}}\cdot \left(V_{m}{-E}_{\mathrm{CaL}}\right){\mathrm{\cdot d}}_{L}{\mathrm{\cdot f}}_{L}{\mathrm{\cdot f}}_{\mathrm{Ca}}$$

$$d_{\mathrm{L,}\infty }\;=1/\left\{1+\exp\left[-\left(V_{m}+13.5\right)/6\right]\right\}$$

$${\text{f}}_{\mathrm{L,}\infty }\;=1/\left\{1+\exp\left[\left(V_{m}+35\right)/7\text{.}3\right]\right\}$$

$$\alpha_{\mathrm{dL}}\;=\;-\text{0}\text{.}02839\cdot \left(V_{m}+\;35\right)/\left\{\exp\left[-\left(V_{m}+\;35\right)/2\text{.}5\right]\;-\;1\right\}\;-\text{0}\text{.}0849\cdot {\text{V}}_{m}/\left[\exp\left(-V_{m}/4\text{.}8\right)-\;1\right]$$

$$\beta_{\mathrm{dL}}\;=\text{ 0}\text{.}01143\text{·}\left(V_{m}\;-\;5\right)/\left\{\exp\left[\left(V_{m}\;-\;5\right)/2\text{.}5\right]-1\right\}$$

$$\tau_{\mathrm{dL}}\;=1/\left(\alpha_{\mathrm{dL}}\;+\;\beta_{\mathrm{dL}}\right)$$

$$\tau_{\mathrm{fL}}\;=\;257\text{.}1\mathrm{\cdot exp}\left\{-{\left[\left(V_{m}+\;32\text{.}5\right)/13\text{.}9\right]}^{2}\right\}+\;44\text{.}3$$

$$f_{\mathrm{Ca,}\infty }\;=K_{\mathrm{mfCa}}/\left(K_{\mathrm{mfCa}}{+\mathrm{Ca}}_{\text{sub}}\right)$$

$$\tau_{\mathrm{fCa}}\;=\;{\text{f}}_{\mathrm{Ca,}\infty }/\alpha_{\mathrm{fCa}}$$

$${dy}_{i}/dt\;=\;\left(y_{\mathrm{i,}\infty }{\;-y}_{i}\right)/\tau_{yi}$$

where, *υ*_*ι*_ *= d*_L_, *f*_L_, *f*_Ca_ are respective channel gating variables of Hodgkin-Huxley type (state variables *y*_*16*_, *y*_*17*_, *y*_*18*_ in Table 2) with steady-state probabilities *d*_*L,∞*_ *, f*_*L,∞*_, *f*_*Ca,∞*_ and gating time constants *τ*_*dL*_, *τ*_*fL*_, *τ*_*fCa*_. *E*_*CaL*_ = 45 mV is an apparent reversal potential of *I*_*CaL*_ and *C*_*m*_ = 32 pF is electric capacitance of the cell membrane.

**FIGURE 3:**
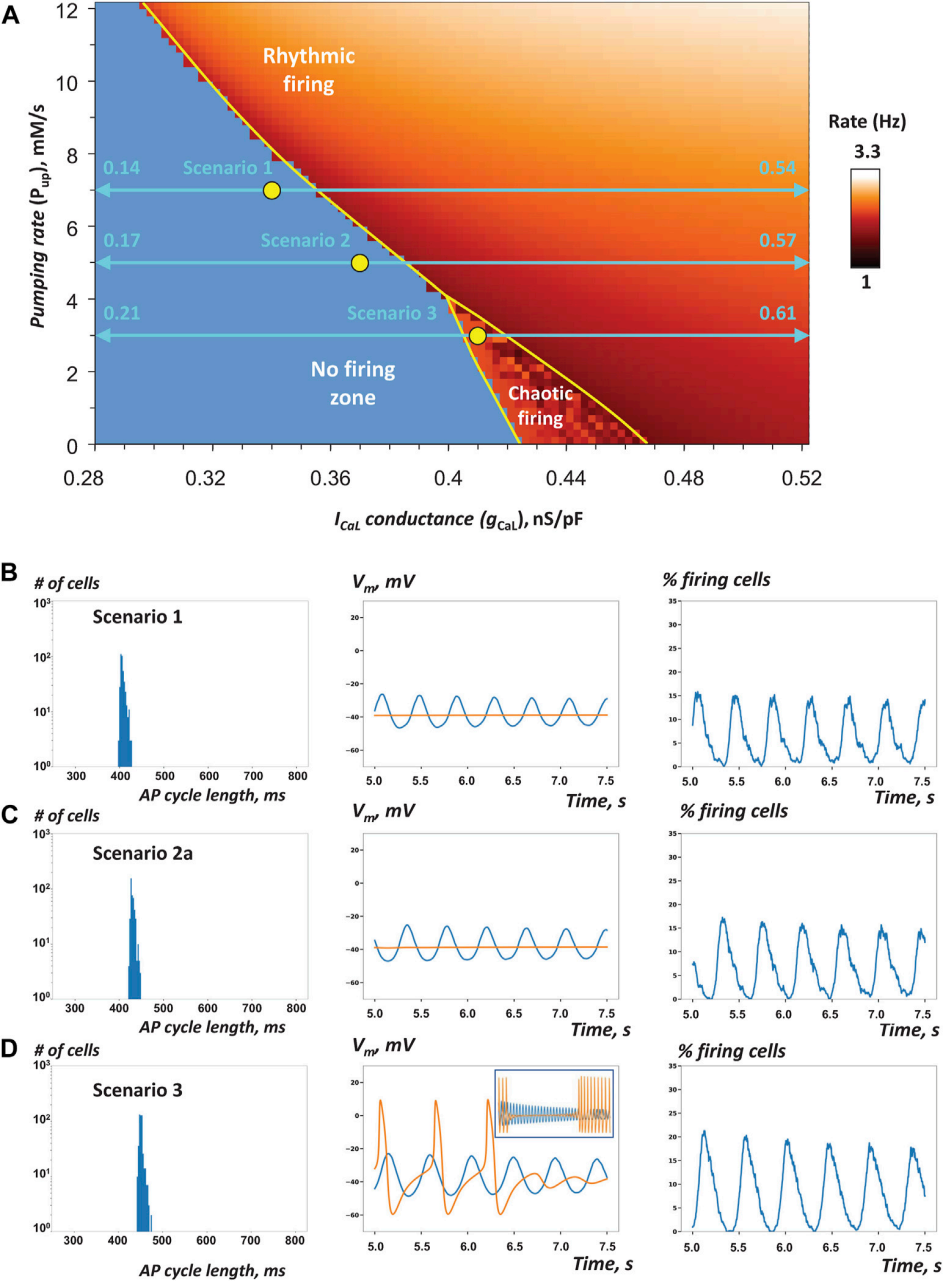
Heterogeneity in *g_CaL_* increases robustness of AP firing in SAN tissue models close to the edge of stability **(A)** *P*_*up*_-*g*_*CaL*_ bifurcation diagram previously reported for single SAN cell model (modified from (Maltsev and Lakatta, 2009)) shows the parametric space for rhythmic AP firing, no firing, and chaotic firing (white labels). Yellow circles show coordinates for fixed *P*_*up*_ and *g*_*CaL*_ mean values (all in the non-firing zone) in cell populations used in our simulations of SAN tissue function in three specific scenarios 1, 2, and 3 (Table 1) close to the bifurcation line (yellow line). Double-headed aqua arrows show the *g*_*CaL*_ ranges for each scenario (Note: the ranges extend beyond the diagram, with the labels showing the extensions). All three tissue models fired rhythmic APs (Supplementary Movies S1–3). **B–D**: Each panel shows the results of simulations in each scenario: histograms of AP cycle length distribution, space-average *V*_*m*_ vs time, and the percentage of firing cells (*V*_*m*_ > 0) vs time (from 5 to 7.5 s). In scenario 3, we extended the simulation run to 25 s. Inset in panel D (middle) shows average *V*_*m*_ from 5 to 25 s. Orange traces show space-average *V*_*m*_ vs time for the control SAN tissue model populated by identical cells with average *P*_*up*_ and *g*_*CaL*_ values of the respective heterogeneous model.

We simulated *I*_*CaL*_ heterogeneity of SAN tissue by varying only *g*_*CaL*_, maintaining all other parameters unchanged. In other terms, only number of functional channels in each cell was varied, but channel gating kinetics remained the same in all cells. We directly linked *g*_*CaL*_ to *I*_*CaL*_ density measured experimentally by whole patch-clamp technique under volage clamp conditions (Figure 1, see details in Results). Once we bridged our model to experimental data, we used a *P*_*up*_ - *g*_*CaL*_ bifurcation diagram previously reported in single cell models (Maltsev and Lakatta, 2009) to guide our selection of *P*_*up*_ and *g*_*CaL*_ distributions for cell populations that fell into parameter-dependent areas of rhythmic firing, chaotic firing, or no firing, bordering at a bifurcation line (split yellow line in Figure 3A). Since our study was focused on the system robustness, we examined the tissue operation in the areas of no firing or chaotic firing (dubbed non-firing zone and chaotic firing zone, respectively) in which individual cells lack normal automaticity but normal pacemaker function is possible in heterogeneous tissue models. The parameters for cell populations were set via a uniformly random distribution within specified ranges (Table 1).

### Algorithm to Create a Cell Cluster

In some model scenarios, we also examined effects of cell clustering within the SAN grid. In each such scenario, one simulation was performed with uniformly random distribution of cells, but the other simulation was performed with the same cell population arranged in a cluster (computer algorithm is provided in Supplementary Material). The algorithm placed the cell with the highest parameter value (*P*_*up*_ or *g*_*CaL*_) exactly at the grid center; the rest of the cell population was sorted in a descending order with respect to each cell parameter value; and the sorted cells were placed spiral-wise around the center. Thus, the last cell with the lowest parameter value was placed at the very periphery. As a result, the algorithm created a cell cluster with smooth decline of the parameter value from center to the periphery.

### Computation, Program Code, Data Analysis and Visualization

Our simulations of SAN tissue function were performed using a new computational approach suggested by Campana (Campana, 2015) based on CUDA technology (Compute Unified Device Architecture). A major advantage of this approach is its parallel processing via GPU that is critical to perform simulations of hundreds or even thousands cell models within SAN tissue within a reasonable time.

The model was originally written in CUDA C programming language for a GPU that featured 64 CUDA cores and 768 MB RAM (Nvidia Quadro FX 1800) (Campana, 2015). Aside from various code optimizations, we adapted the code to modern, high-performance GPUs such as the TITAN RTX graphics card (NVIDIA corp. CA, USA) featuring 4608 CUDA cores and 24 GB RAM used in the present study. One specific important improvement was that we arranged CUDA blocks in a grid, rather than in a one-dimensional array in the original code. Furthermore, random numbers were properly initialized in the main function of Central Processing Unit and copied to GPU memory to generate random values for *g*_*CaL*_ and *P*_*up*_ for each cell. On average, one 7.5 s simulation of SAN tissue function took about 15 min of computation time.

All simulations began with the same initial conditions, near to the maximum diastolic potential (Table 2). In nearly all simulations, the system reached a steady pattern of AP firing (or no firing) in about 5 s. Thus, our standard simulations lasted 7.5 s, and we examined the system behaviors in the last 2.5 s. Some simulations were run longer, for 25s, when the system required more time to reach its steady AP firing pattern. The data analysis and visualizations (including movies of *V*_*m*_ in the tissue) were performed using in-house written programs in Python 3.10.1 (Python Software Foundation, www.python.org) and Delphi 10.4. (Embarcadero, Austin, TX). AP cycles were detected by a modified algorithm of Python library SciPy: scipy.signal.find_peaks https://docs.scipy.org/doc/scipy-1.1.0/reference/generated/scipy.signal.find_peaks.html.

All histograms of AP cycle length were shown on a semi-logarithmic scale to better illustrate and compare AP firing synchronization in various contexts.

## Results

### Matching the Experimentally Measured *I*_*CaL*_ Densities and the Model Parameter *g*_*CaL*_

To bridge realistic *I*_*CaL*_ densities (measured in pA/pF by patch clamp (Monfredi et al., 2018), Figure 1A) and the model parameter, *g*_*CaL*_ (in nS/pF), we performed voltage-clamp simulations of our single cell model (Figure 1B) with the voltage step protocol (see inset) similar to that used in experimental studies (Honjo et al., 1996; Monfredi et al., 2017). The *I*_*CaL*_ - *V*_*m*_ relationship for the peak values of the simulated *I*_*CaL*_ traces (Figure 1C) revealed a maximum peak *I*_*CaL*_ current of 9.068 pA/pF at 0 mV with a basal state conductance of 0.464 nS/pA, bridging experimental and model data via black dash line in the dual Y scale plot in Figure 1A. In our tissue model scenarios involving a large variety of *g*_*CaL*_ values in individual cell models, we always referred to Figure 1A to make sure that *g*_*CaL*_ remained within the respective range of *I*_*CaL*_ densities measured experimentally.

### SAN Models With Heterogeneous *g*_*CaL*_ Close to the Bifurcation Line

First, we tested the hypothesis that reported heterogeneity in *I*_*CaL*_ density among SAN cells (i.e. *g*_*CaL*_ in the model terms) adds robustness to AP firing within SAN tissue. We constructed scenarios in which the mean *g*_*CaL*_ values were in the non-firing zone along the bifurcation line (yellow circles in Figure 3A), but with a substantial spread of individual *g*_*CaL*_ values (horizontal double-headed aqua arrows). Three scenarios with different *g*_*CaL*_ distributions were tested to ensure robustness of the simulation results. Our specific choice of *g*_*CaL*_ range in each scenario is given in Table 1 (scenarios 1–3).

While each cell in the tissue is represented by a deterministic model of ordinary differential equations, *g*_*CaL*_ in each cell was assigned by a random number generator within a specified range to mimic tissue heterogeneity. Therefore, we addressed possible effects of randomness in *g*_*CaL*_ distribution by running three simulations for scenario 2 with the same model parameters, but with different seed values for random number generator (2a, 2b, 2c in Table 1).

Our simulation results for scenarios one to three are presented in Figure 3B–D and Table 1, and visualized in Supplementary Movies S1–3. The percentage of AP firing cells within the time interval from 5 to 7.5 s after simulation onset was the most important parameter, by which SAN tissue performance was evaluated (columns “% firing cells” tissue vs separate in Table 1).

In all three scenarios, rhythmic AP firing was indeed observed in our simulations of SAN heterogeneous models (Figure 3B–D, blue lines), whereas all models populated with identical cells failed to generate APs (Figure 3B–D, orange lines in middle panels). It is also important to note that percentage of firing cells in heterogenous tissue was higher than percentage of firing cells firing separately (Table 1). In scenarios 1 and 2, impulses originating within multiple areas in the grid spread in a wave-like fashion to other areas involving the majority of cells (Supplementary Movies S1, S2A–C). In scenario 3, rhythmic wave-like AP propagation recruited almost all cells (83.2%, histogram in Figure 3D, Supplementary Movies S3), despite mean values of *P*_*up*_ and *g*_*CaL*_ fell into the chaotic firing zone (Figure 3A). For scenario 3, we extended our simulation to 25 s to ensure that the control model populated with identical cells indeed generated chaotic firing (orange line in Figure 3D, middle panel inset), rather than just ceased firing after 7.5 s in our shorter standard simulation run. Thus, scenario 3 explicitly shows that adding heterogeneity to *g*_*CaL*_ can have **antiarrhythmic** effect: dysrhythmic (chaotic) firing in SAN tissue model populated by identical cells is shifted to rhythmic firing in the heterogeneous SAN model.

Histograms of AP firing length distribution calculated for all three scenarios (Figure 3B-D, left panels) exhibited sharp peaks between 400 and 500 ms with a relatively small standard deviation of about 10 ms (Table 1). Synchronization of AP firing among cells is also evident from grid-average *V*_*m*_, oscillation amplitude (about 25 mV, middle panels) and the percentage of firing cells (*V*_*m*_ > 0) that oscillated with an amplitude of 15–20% (right panels).

**FIGURE 4:**
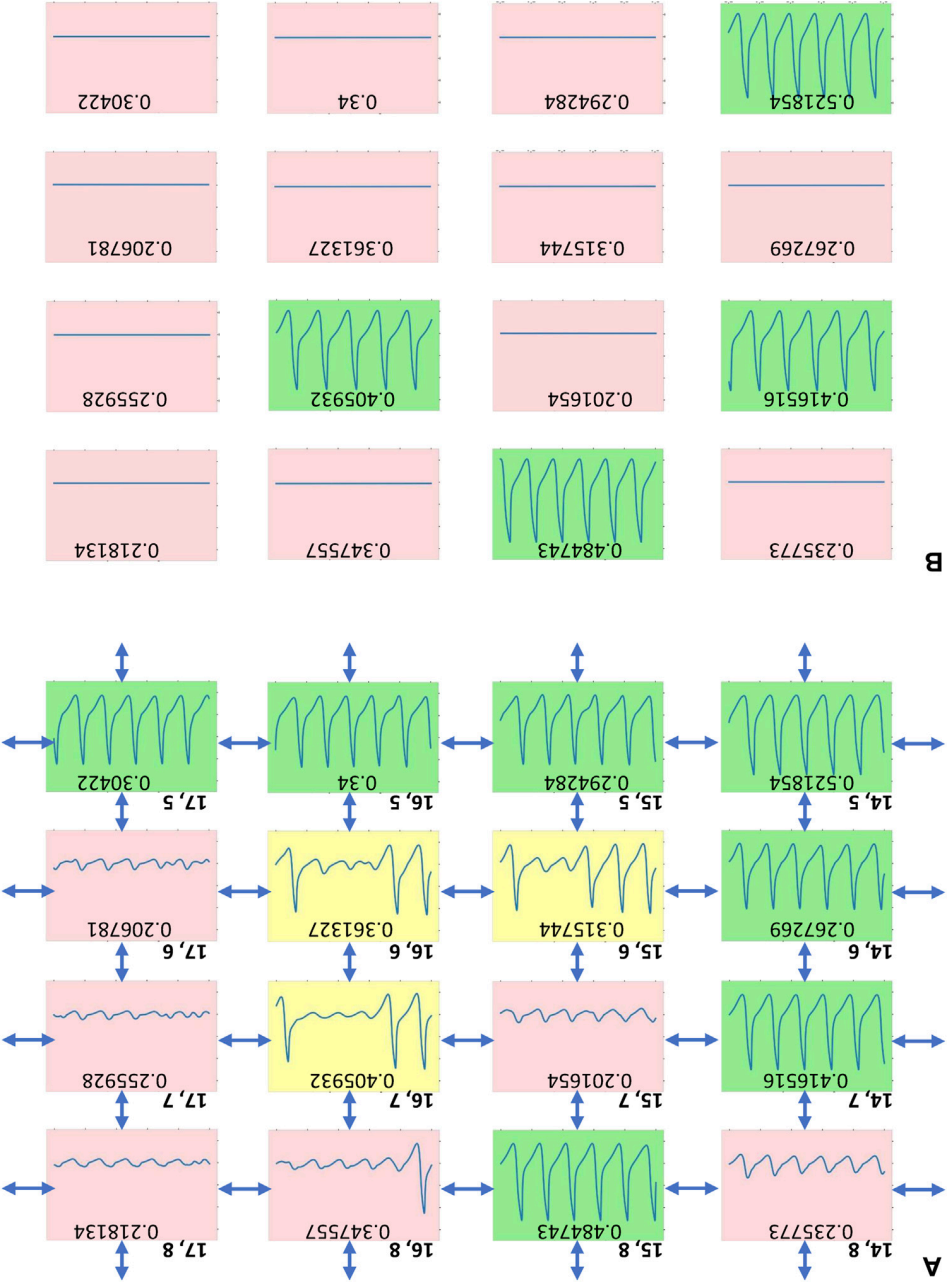
Emergence of chaotic firing in cell tissue with heterogenous *g_CaL_* distribution **(A)** AP firing in a tissue fragment with a local community of three chaotically firing cells in scenario 2c (Table 1, Supplementary Movies S2C) **(B)** the same tissue fragment when cells are not connected. Each cell is presented by its 2.5 s *V*_*m*_ time series (from 5 to 7.5 s after simulation onset) in a multicolor box designating cell operation mode: green - rhythmic AP firing; pink—no firing (subthreshold *V*_*m*_ oscillations); and yellow—chaotic firing. Double headed arrows designate cell connections. *g*_*CaL*_ value of each cell model (in nS/pF) is shown at the top of its time series. Cell coordinates (x,y) within the tissue grid are shown in the left top corner of each subpanel. The coordinates (1,1) are set for the cell in the left bottom corner in Supplementary Movies S2C. The *V*_*m*_ scale in each subpanel is from -70 to 30 mV.

All three simulation runs (2a, 2b, 2c in Table 1) for *g*_*CaL*_ distribution with different seeds of random number generator exhibited, in general, well synchronized AP firing with approximately the same percentage of firing cells and average cycle length, although with substantially different standard deviations. A closer inspection of AP firing within the tissue revealed a minor population of cells in runs 2b and 2c that generated substantially longer cycle lengths that caused this difference. These cells formed clusters of chaotically firing cells (Figure 4A), whereas the same cells in isolation (Figure 4B) exhibited only rhythmic firing or no firing. Thus, the new chaotic firing behavior emerges within the cell cluster from complex interactions of cells with intrinsic automaticity and dormant cells.

### SAN Models With Heterogenous *P*_*up*_

**FIGURE 5:**
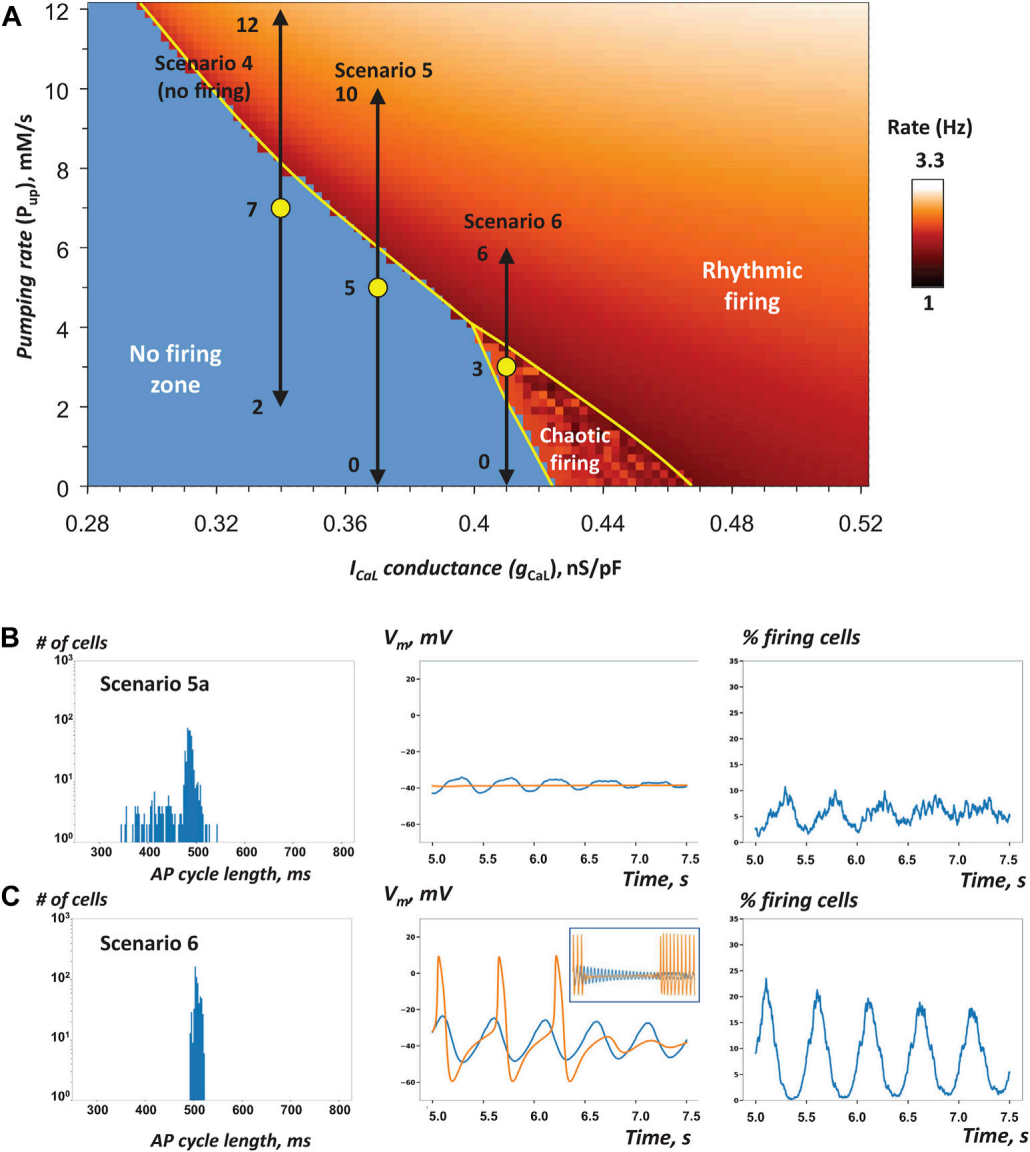
Heterogeneity in *P_up_* increases robustness of AP firing in SAN tissue models close to the edge of stability **(A)** *P*_*up*_-*g*_*CaL*_ bifurcation diagram illustrating cell populations in simulations of SAN tissue function in scenarios 4, 5, and 6 (Table 1) similar to Figure 3A. Yellow circles show coordinates for mean *P*_*up*_ and fixed *g*_*CaL*_ values (in non-firing and the chaotic firing zones). Black double-headed arrows show the exact *P*_*up*_ ranges for each scenario. The tissue model with low *g*_*CaL*_ in scenario 4 failed to generate AP firing despite a higher average *P*_*up*_ value (Supplementary Movie S4), but scenarios 5a and 6 with higher *g*_*CaL*_ values fired rhythmic AP (Supplementary Movies S5A, S6). **B–C**: the results of simulations in scenarios 5a and 6. In scenario 6, we extended the simulation run to 25 s. Inset in panel C (middle) shows average *V*_*m*_ from 5 to 25 s.

Next, we examined three model scenarios (4, 5, 6 in Table 1) in which *P*_*up*_ was uniformly randomly distributed, but *g*_*CaL*_ was fixed to a certain value in each scenario. The *P*_*up*_ value in a given cell mimics the functional effect of phospholamban phosphorylation to regulate SR Ca pumping via SERCA2a. The mean values of *P*_*up*_ and fixed *g*_*CaL*_ (yellow circles in Figure 5A) were chosen to move along and close to the bifurcation line, still remaining within the non-firing zone. Here we tested the specific hypothesis that adding heterogeneity to *P*_*up*_ in cell populating SAN tissue increases its robustness to generate rhythmic AP firing.

A substantial cell-to-cell variability in phospholamban phosphorylation is expected based on a substantial variability of both basal AP firing rates and responses to β-adrenergic stimulation among isolated SAN cells (Kim et al., 2021). However, in contrast to *g*_*CaL*_, there is no single-cell data in the literature to set up cell populations in the model within an exact *P*_*up*_ range. To get an idea of what to expect for physiologically relevant lower limit in *P*_*up*_ variations in individual cells (when testing model stability), one can refer, for example, to how low *P*_*up*_ can descend during the physiological responses to cholinergic receptor stimulation. Within physiologic range of acetylcholine concentrations up to 1 μM, the *P*_*up*_ values can decrease on average from 12 mM/s to as low as 4 mM/s (Maltsev and Lakatta, 2010), indicating that *P*_*up*_ can actually be very flexible in each SAN cell during its normal operation. Thus, we constructed and tested three model scenarios with substantial variability of *P*_*up*_ (Figure 5; Table 1, scenarios 4–6; Supplementary Movies S4–6). Similar to simulations with *g*_*CaL*_, we also addressed possible effects of randomness in *P*_*up*_ distribution by running three simulations for scenario 5 with the same model parameters, but with different seed values for random number generator (5a, 5b, 5c in Table 1).

Our model simulations in scenarios 5a, 5c, and 6 demonstrated that *P*_*up*_ heterogeneity can indeed restore SAN tissue operation within the non-firing zone, thus supporting our hypothesis. In scenario 4, however, *g*_*CaL*_ (and hence cell excitability) were set so low, that none of the 625 cells in the entire grid continued to generate APs after about 4 s (Supplementary Movie S4). As *g*_*CaL*_ increased in scenarios 5 and 6, the tissue generated APs, despite the decreasing average of *P*_*up*_ values (Table 1). This indicates importance of *g*_*CaL*_ and clock coupling in this mechanism of robustness. Surprisingly and in contrast to simulations involving the *g*_*CaL*_ spread in the previous section, all or almost all cells fired rhythmic APs (Table 1). However, the tissue-wide AP firing occurred in a less synchronized manner, especially in scenario 5 (runs 5a, 5b, 5c), manifested by a larger spread in AP cycle lengths among cells (histogram in Figure 5B, large SD values in Table 1, Supplementary Movies S5A–C). The percentage of firing cells at a given time was extremely noisy. Some cells formed local communities with unsynchronized AP firing, including chaotically firing cells and low amplitude sub-threshold oscillations (Supplementary Figure S1). Moreover, scenario 5b ceased firing at about 6 s, yielding only about half cells firing (44.8%) during evaluation period (from 5 to 7.5s), while scenarios 5a and 5c showed almost all cells firing APs (97.9 and 99.8%), indicating that a particular distribution (at a given seed value) is indeed important when the system operates at the edge of stability.

### Rescuing AP Firing Requires Substantial Heterogeneity in *P*_*up*_ or *g*_*CaL*_

**FIGURE 6:**
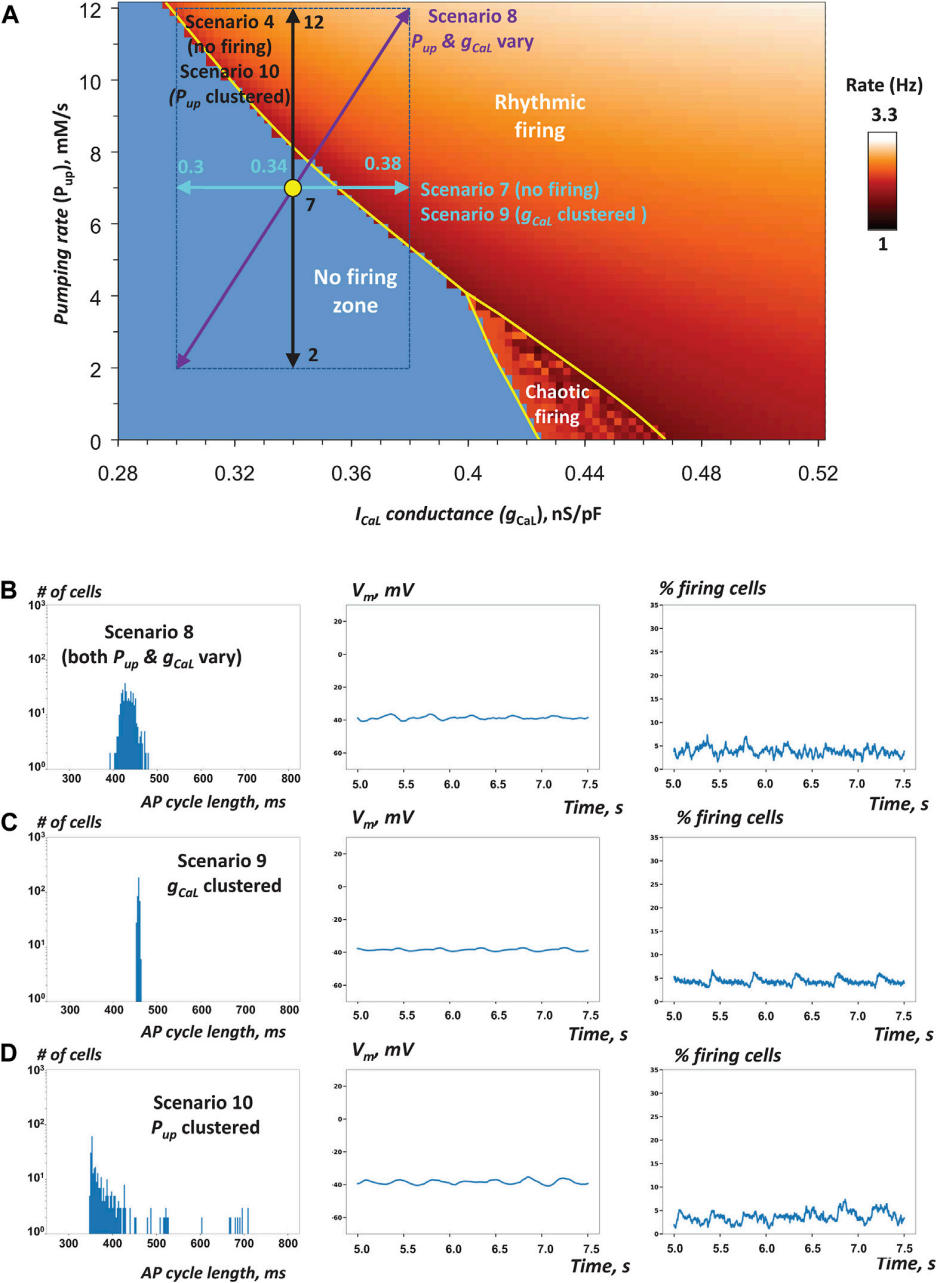
Robustness of SAN operation is increased via combined *P_up_* and *g_CaL_* heterogeneities and their clustering **(A)** *P*_*up*_-*g*_*CaL*_ bifurcation diagram illustrating cell populations in simulations of SAN tissue function in scenarios 7–10 (Table 1) similar to Figures 3A, 5A. Yellow circle shows coordinates for mean *P*_*up*_ and mean *g*_*CaL*_ values (in the non-firing zone). Double-headed arrows show *P*_*up*_ and *g*_*CaL*_ ranges in these scenarios. The tissue models with heterogeneity in either single parameter failed to generate rhythmic APs (Supplementary Movies S4, 7). However, when both parameters fluctuated, the tissue model fired rhythmic APs (Supplementary Movie S8). Furthermore, when either parameter (*g*_*CaL*_ or *P*_*up*_) was clustered, the tissue also generated APs in scenarios 9 or 10 (Supplementary Movies S9, 10), respectively. **B–D**: the results of simulations in scenarios 8–10.

It is important to emphasize that in all above scenarios rescuing AP firing required a substantial spread of *g*_*CaL*_ or *P*_*up*_ values. In other terms, in the non-firing zone at each *P*_*up*_ value, the spread of *g*_*CaL*_ must reach a critical value in order to rescue AP firing. While the spread of 0.4 nS/pF is indeed substantial, it was realistic, because assigned *g*_*CaL*_ values remained within experimentally measured range of *I*_*CaL*_ as shown in our bridging dual Y plot in Figure 1A. We tested several additional scenarios with narrower *g*_*CaL*_ distribution spreads <0.4 nS/pF, but those non-realistic SAN tissue models were unable to converge to steady AP firing. One such failed tissue model is shown in Figure 6A (scenario 7, no firing) by aqua double-headed arrow (see also Supplementary Movie S7).

### Functional Synergy of *P*_*up*_ and *g*_*CaL*_ Heterogeneities

To gain further insights into SAN tissue operation, we tested the hypothesis that heterogeneity of cell populations with respect to both Ca clock and membrane clock would act synergistically to increase robustness of the pacemaker function. When *P*_*up*_ or *g*_*CaL*_ were distributed separately in scenarios 4 and 7 (described above), the respective SAN tissue models lacked automaticity (Supplementary Movies S4, S7). Thus, we simulated and examined an additional special scenario 8 that included the distributions of both *g*_*CaL*_ and *P*_*up*_ assigned in scenarios 4 and 7 (purple diagonal double-headed arrow in Figure 6A and Table 1). Our simulations showed that such a system with dual parameter distribution generated APs (Figure 6B, Supplementary Movie S8), indicating that heterogeneities in *g*_*CaL*_ and *P*_*up*_ act indeed synergistically in rescuing normal system operation within the non-firing zone. Remarkably, the percentage of firing cells in the tissue increased to 69% from 42% for the same cell population when cells are not connected to each other, indicating ongoing recruitment of dormant cells to fire APs when cells were connected. AP firing in this scenario, however, was poorly synchronized among cells with such narrow distribution of *g*_*CaL*_ values around a low mean value of only 0.34 nS/pF. Desynchronized cells included chaotically firing cells and low amplitude sub-threshold oscillations (Supplementary Figure S2).

### *P*_*up*_ or *g*_*CaL*_ Clustering Is yet Another Mechanism of Robust SAN Function

In all previous scenarios *g*_*CaL*_ and *P*_*up*_ were uniformly randomly distributed within a specific range. Experimental studies, however, indicate that the SAN features clusters of cells with different types of activity (Figure 4 in Bychkov et al., 2020). Thus, we tested the hypothesis that the natural cell clustering provides an additional “gear” to enhance robustness of AP firing in SAN tissue. To test this hypothesis, we attempted to rescue automaticity in scenario 7 in which cells had heterogeneous *g*_*CaL*_ within an extremely narrow range from 0.3 to 0.38 nS/pF. Indeed, when cells were uniformly randomly distributed over the tissue, the modelled system of cells lacked automaticity However, the system was able to achieve rhythmic AP firing when the same heterogenous cell population was locally redistributed with higher *g*_*CaL*_ clustering towards the grid center (Figure 6C; Table 1, scenario 9; Supplementary Movie S9). Finally, we tested the hypothesis that, similar to *g*_*CaL*_, increased heterogeneity in *P*_*up*_ can also add robustness to the system of interacting cells. Indeed, while the tissue model in scenario 4 lacked automaticity with uniformly random *P*_*up*_ distribution among cells within the tissue (Supplementary Movie S4), clustering cells with higher *P*_*up*_ towards the tissue center rescued the system automaticity (scenario 10, Figure 6D and Table 1, Supplementary Movie S10). It is important to note that in both simulations with *g*_*CaL*_ and *P*_*up*_ clustering the AP firing was limited to grid center and lacked at the grid border. Chaotically firing cells and low amplitude sub-threshold oscillations were observed at the cluster periphery (at the border of firing and non-firing cells) in simulations with *P*_*up*_ distribution (Supplementary Figure S3).

### Effects Cell-To-Cell Coupling Strength at the Edge of the System Stability

The model parameter determining cell-to-cell coupling strength is ρ that is the intracellular resistivity (the lower resistivity, the stronger coupling). In our simulations thus far, ρ was set to the highest value of 10^4^ MΩ·m of the original Campana model (Campana, 2015) to reflect low cell coupling known for the central part of the SAN where the cardiac impulse originates (Boyett et al., 2006). Campana examined heterogeneous SAN tissue with average parameter values exactly as in original Maltsev-Lakatta model with *g*_*CaL*_ = 0.464 nS/pF and *P*_*up*_ = 12 mM/s, i.e. in rhythmic firing zone, very far from the bifurcation line, and found notable effects of cell coupling when ρ changed by orders of magnitude, i.e. 10^4^, 10^3^, 10^2^, 10, and 1 MΩ·m. Campana’s studies showed that the mean AP cycle length increased (i.e. the rate decreased) as the coupling strength increased and when coupling was extremely strong (*ρ* = 1 MΩ·m) the AP cycle length approached the average cycle length of isolated cells, i.e. ∼333 ms of the original Maltsev-Lakatta model. In contrast to Campana’s simulations, our scenarios were chosen in non-firing zone, and what may happen with such system operating at the edge of stability at different coupling strength is not obvious and may provide insights into robust operation of SAN.

**FIGURE 7:**
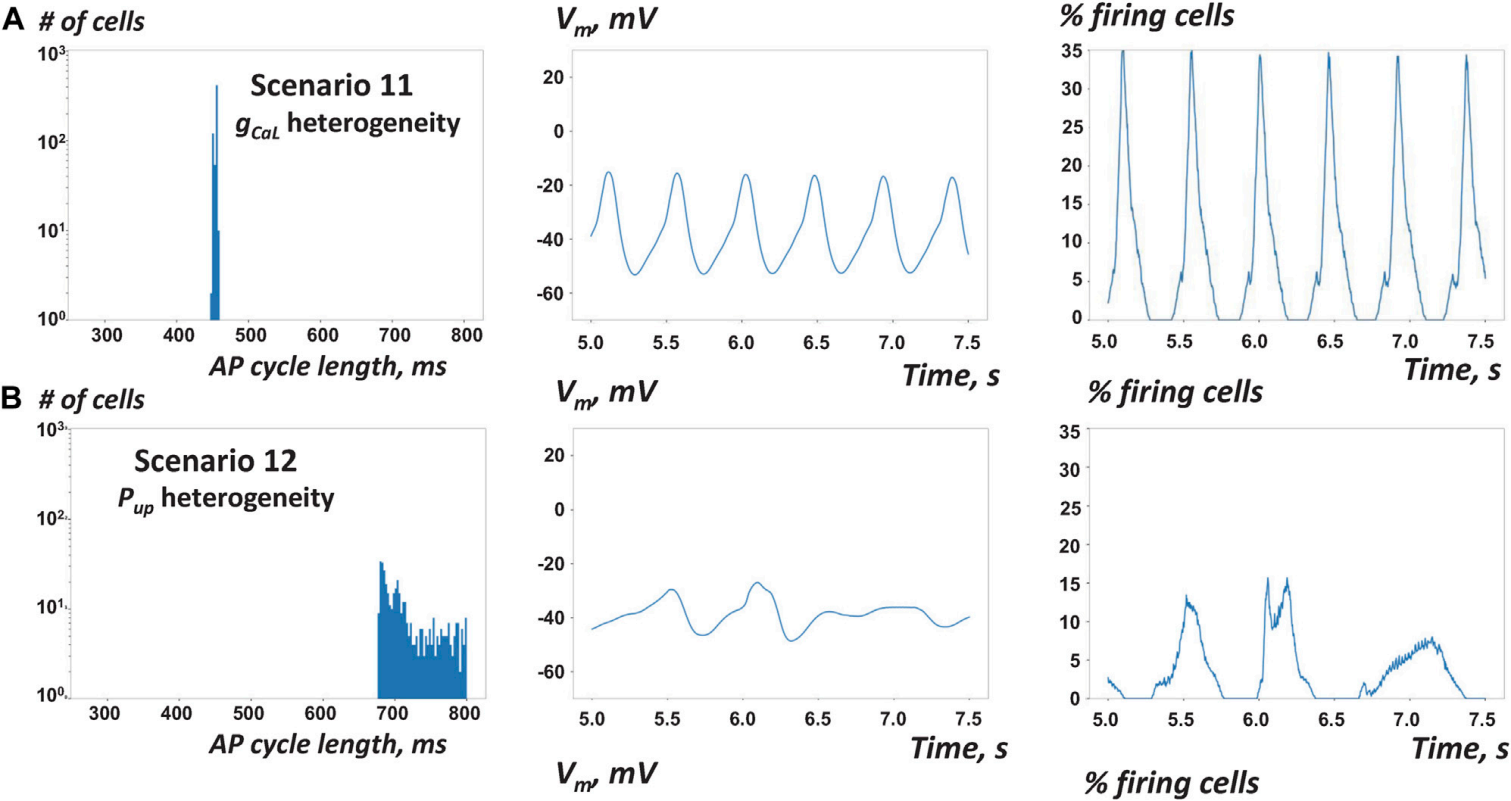
Results of simulations of SAN tissue function with stronger cell-to-cell coupling **(A)** With stronger cell coupling at ρ = 10^3^ MΩ·m vs 10^4^ MΩ·m (scenario 11 vs 2) AP firing in tissue with heterogeneous *g*_*CaL*_ became slightly slower, but more synchronized (almost metronomic, Supplementary Movie S11) **(B)** With stronger cell coupling in tissue with heterogeneous *P*_*up*_ (scenario 12 vs 5) AP firing in tissue with heterogeneous *P*_*up*_ became substantially slower and remained notably unsynchronized.

To this end, here we performed additional simulations with new settings of ρ for scenario two featuring *g*_*CaL*_ heterogeneity and scenario 5 featuring *P*_*up*_ heterogeneity. We set ρ to intermediate coupling (*ρ* = 10^3^ MΩ·m, scenarios 11 and 12) and very strong coupling (*ρ* = 1 MΩ·m, scenarios 13 and 14). In scenarios 11 and 12 with intermediate coupling, the average AP cycle length substantially increased (Figure 7; Table 1, Supplementary Movies S11, 12) vs respective scenarios 2 and 5 with weakly connected cells at *ρ* = 10^4^ MΩ·m (Figures 3C, 5B) consistent with Campana’s results. However, in contrast to Campana’s results, in scenarios with strong coupling, the SAN did not settle at an average cycle length of isolated cells, but ceased function (Table 1, scenarios 13 and 14, Supplementary Movies S13, 14).

### Effects of Cell Heterogeneity With the Average Parameter Values in the Rhythmic Zone of the *P*_*up*_ *- g*_*CaL*_ Diagram

All our previously tested scenarios were performed within the “no firing” zone to examine the system robustness at the border of system stability. However, for accurate comparison, it is also important to run control simulations and examine how the system behaves on the other side of the bifurcation line, in the rhythmic zone. These control simulations ought to exclude possibility that dormant cells (still present in the heterogeneous cell population in the firing zone) do not halt SAN automaticity. To this aim, we constructed four additional model scenarios, in which we shifted either *g*_*CaL*_ or *P*_*up*_ towards rhythmic zone. (Table 1, Scenarios 15–18, Supplementary Movies S15–18).

These control simulations showed that the SAN tissue populated by cells with their average parameter values in rhythmic zone indeed generates rhythmic APs as expected. An important result of these simulations was that the system of interacting heterogeneous cells operates differently (in a more robust way) *vs*. the population of same cells with no interactions: the percentage of firing cells within the system is higher than the percentage of cells with intrinsic automaticity (Table 1). In other words, intrinsically firing cells recruit to fire many dormant cells within the functional SAN tissue.

## Discussion

### Our New Approach to the Problem

Heterogeneity of SAN cells has been known and well-documented for a long time (Boyett et al., 2000), but its importance for SAN function remains unclear. Numerical model simulations performed in the present study revealed an interesting and counterintuitive aspects of the problem: While noise and heterogeneity are usually associated with undesirable disturbances or fluctuations, SAN cell heterogeneity can increase robustness of cardiac pacemaker function. Furthermore, while stronger cell connections seem to lead to higher synchronization and better function, robust function of heterogeneous SAN tissue actually requires weak cell coupling but stronger cell coupling at the edge of stability leads to SAN failure. Our work took advantage of well-described properties of single isolated SAN cells in numerous previous experimental and theoretical studies. Specifically, the intrinsic behaviors of individual cells have been characterized by parametric sensitivity analyses (Maltsev and Lakatta, 2009; Kurata et al., 2012) that revealed synergistic contributions of the two key parameters *P*_*up*_ and *g*_*CaL*_ to generate normal automaticity in individual SAN cells as depicted in *P*_*up*_ - *g*_*CaL*_ diagram (panel A in Figures 3,5,6). This diagram was helpful to guide the model simulations (Table 1) and interpret our results. We investigated how the SAN tissue behaves when it is populated by a large variety of individual cells with known intrinsic properties (i.e. the systems approach). On the other hand, our consideration was limited to only two key model parameters *g*_*CaL*_ and *P*_*up*_ and their combinations (i.e. we combined reductionist and systems approaches).

### Comparison With Previous Numerical Studies of SAN Tissue

A plethora of numerical models of SAN tissue function have been developed previously, including models in one dimension (Zhang et al., 2001; Huang et al., 2011; Glynn et al., 2014; Li et al., 2018), two dimensions (Michaels et al., 1987; Oren and Clancy, 2010; Campana, 2015; Gratz et al., 2018; Karpaev et al., 2018), and full-scale SAN models in three dimensions (Munoz et al., 2011; Li et al., 2013; Li et al., 2014; Kharche et al., 2017; Mata et al., 2019). These models examined effects of cell-to-cell coupling (Li et al., 2013; Campana, 2015; Gratz et al., 2018; Mata et al., 2019), inter-cellular variability (Campana, 2015; Mata et al., 2019), coupling to other cell types (fibroblasts) (Oren and Clancy, 2010; Karpaev et al., 2018), and normal and abnormal impulse propagation in three dimensions (Munoz et al., 2011; Li et al., 2014; Kharche et al., 2017).

To our knowledge only two numerical studies (Campana, 2015; Mata et al., 2019) have investigated the impact of heterogeneity of cell parameters on SAN tissue function. However, in both studies cell heterogeneity was limited to only surface membrane clock parameters and mimicked by a simultaneous randomization of all membrane conductances with respect to their basal values within a fixed percentage range (dubbed sigma). Mata et al. (Mata et al., 2019) reported time course of synchronization of AP firing vs sigma. Campana (Campana, 2015) reported that the cell population synchronizes on a rate slightly higher than the one of the isolated cells, but it does not equal the rate of the fastest cell. Here we investigated importance of heterogeneity not only with respect to ion currents, but also Ca clock function. Furthermore, we examined the effects of tissue heterogeneity at the edge of the system stability in the non-firing zone, critical for robust function of SAN tissue.

Examining Ca signals in addition and together with membrane clock parameters is vital in studies of pacemaker function because each SAN cell operates as a coupled-clock system, i.e. actions of *I*_*CaL*_ and *P*_*up*_ are intertwined in a synergistic manner in numerical models of both isolated cells (Maltsev and Lakatta, 2009; Kurata et al., 2012) and SAN tissue (Figure 6A,B). Among membrane currents, we chose *I*_*CaL*_ for our analysis because it is involved not only in membrane clock function (it generates AP upstroke (Mangoni and Nargeot, 2008), but also in clock coupling by providing influx of Ca (coupled clock’s oscillatory substrate) (Maltsev and Lakatta, 2009; Lakatta et al., 2010) and via positive feedback mechanisms among local Ca releases, Na/Ca exchanger, and *I*_*CaL*_ during diastolic depolarization (Torrente et al., 2016; Lyashkov et al., 2018). Distributions of *I*_*CaL*_ among individual SAN cells have been reported in previous experimental studies (Honjo et al., 1996; Musa et al., 2002; Monfredi et al., 2017) and thus permitted realistic choices for our *I*_*CaL*_ modeling (Figure 1A).

Recent theoretical studies have demonstrated that the presence of a Ca clock in addition to a membrane clock protects the SAN from annihilation, associated with sinus node arrest (Li et al., 2018); and that random parameter heterogeneity among oscillators can consistently rescue the system from losing synchrony (Zhang et al., 2021). These prior reports and the results of the present study support a novel SAN structure/function paradigm (resembling neuronal networks) that was proposed based on high-resolution imaging of SAN tissue (Bychkov et al., 2020): in the new paradigm, interactions of Ca signals and APs play a central role in generation of rhythmic cardiac impulses that emanate from the SAN.

### The Coupled-Clock Theory Explains, in Part, the Effect of Heterogenous Cell Populations to Enhance Robustness of SAN Function

The coupled-clock theory (Maltsev and Lakatta, 2009) postulates that the intrinsic cell automaticity is defined by a combined and synergistic action of the Ca and membrane clocks as depicted in the *P*_*up*_-*g*_*CaL*_ diagram (Maltsev and Lakatta, 2009) (panels A in Figures 3,5,6). Also, perturbation of ether clock inevitably affects the other clock and the entire coupled-clock system (Yaniv et al., 2013), explaining, in part, our result that the robustness of AP firing in SAN tissue can be enhanced when either *g*_*CaL*_ or *P*_*up*_ varies among the cells (Figures 3, 5; Table 1).

More specifically, by allowing *P*_*up*_ and/or *g*_*CaL*_ to substantially deviate from their mean values, we actually create a sub-population of cells that have intrinsic automaticity in addition to the sub-population of intrinsically non-firing cells (dubbed dormant cells (Kim et al., 2018; Tsutsui et al., 2018; Tsutsui et al., 2021). The two sub-populations of cells intimately interact, determining the ultimate performance (or failure) of the SAN tissue they comprise. Thus, the coupled-clock theory explains, in part, another important result of our study that *g*_*CaL*_ and *P*_*up*_ heterogeneities act synergistically in rescuing SAN from failure. When the SAN system featured heterogeneity in both *g*_*CaL*_ and *P*_*up*_, almost all cells (∼70%, scenario 8) generated APs in marked contrast to SAN tissue with heterogeneity in either *g*_*CaL*_ or *P*_*up*_, in which all cells remain non-firing (scenarios 4 and 7). Furthermore, the fact that the SR Ca pumping is a key timing mechanism in the coupled-clock theory can also explain the differences in rescuing tissue automaticity via *P*_*up*_ or *g*_*CaL*_: AP cycle length substantially varied in tissues with *P*_*up*_ heterogeneity (scenario 5), whereas only slight AP cycle length variability was observed in tissues with *g*_*CaL*_ heterogeneity (scenarios 1–3).

### Emergence of SAN Tissue Automaticity Is a Critical Phenomenon

**FIGURE 8:**
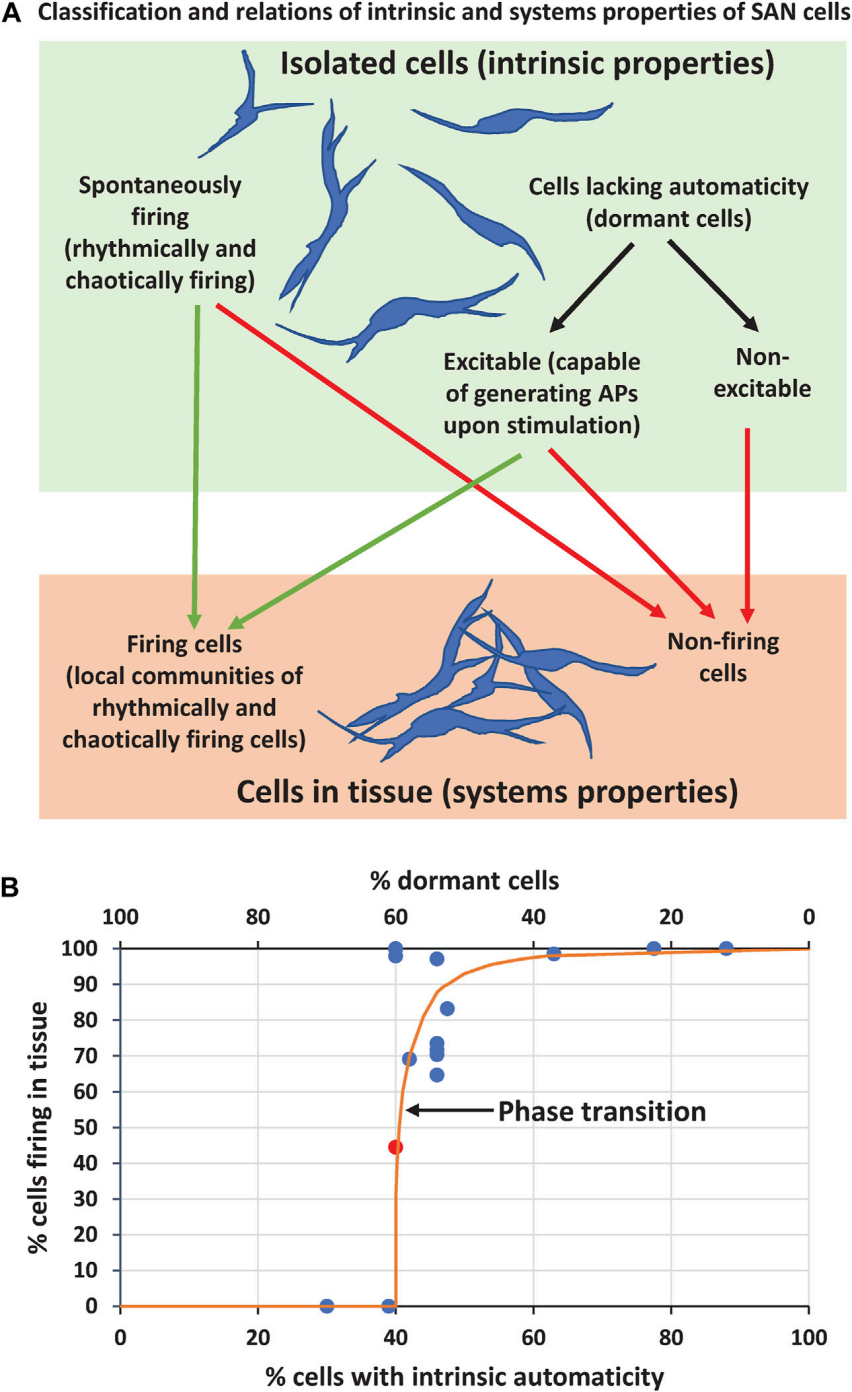
Results summary and interpretation **(A)** Classification of intrinsic properties of individual SAN cells and their relation to properties of SAN cells within tissue **(B)** A summary graph based on data in Table 1 (except scenarios 9, 10, 13, 14) showing relation of the percentage of firing cells in tissue model as the function of percentage of intrinsically firing cells (bottom *x* axis) or dormant cells (top *x* axis). Orange line illustrates a hypothetical phase transition (free-hand line with no theoretical value). Note that cell tissue can function with the percentage of intrinsically firing cells as low as 40% (i.e. 60% of dormant cells). Red circle in the middle of the plot shows the data point for scenario 5b reflecting an “intermediate” system performance during its phase transition from firing to non-firing state (AP firing ceased at about 6 s, see Supplementary Movie S5B).

While the coupled-clock theory can explain, in part, some results of the present study, its application to tissue function is limited, because it predicts only intrinsic cell properties, i.e. cell operation in isolation. Cell tissue, however, operates at a higher level of organization and its automaticity emerges from interactions of numerous cells with different intrinsic properties within local cell communities. These interactions go beyond the coupled-clock theory (at least in its present form). Let’s classify intrinsic properties of the cells and examine how they operate within the system (Figure 8A).

Parameter *g*_*CaL*_ is critical for cell excitability because *I*_*CaL*_ generates AP upstroke. Therefore, in our scenarios with substantial *g*_*CaL*_ spread (including very low values, like in real SAN cells, Figure 1A), the entire cell population can be split into two major sub-populations with respect to cell intrinsic properties: 1) spontaneously firing APs and 2) cells lacking automaticity (dormant cells). In turn, each sub-population can be further split into two categories: spontaneously firing cells can be rhythmically firing or chaotically firing; cells lacking automaticity can be excitable (capable of generating APs upon stimulation) or non-excitable if *g*_*CaL*_ is too low to generate an AP. This classification helps to understand and interpret our simulation results with respect to emergence of automaticity in SAN tissue. For example, non-excitable cells will always remain non-firing either in isolation or in tissue, whereas excitable cells can be recruited to fire APs, but spontaneously firing cells can cease firing in the tissue (Figure 8A).

One important result of our study is that the percentage of firing cells in tissue in all scenarios involving either *P*_*up*_ or *g*_*CaL*_ (except scenarios lacking automaticity) was much larger than that when the same cells operated in isolation (Table 1), indicating that some excitable cells lacking automaticity were indeed recruited to fire APs by their neighboring cells in local cell communities to increase overall performance of the SAN tissue. And vice versa, the SAN tissue surprisingly lacked automaticity in scenarios 4 or 7, despite a major fraction of cells (39% or 30%, respectively, Table 1) was capable of generating rhythmic APs, according to the coupled-clock theory, but did not fire APs in the tissue.

Thus, our simulations at the edge of the system stability discovered strong amplification (i.e. self-organization) of either AP firing or strong suppression of AP firing among the cells within SAN tissue. In other terms, whether the system gains automaticity or fails, seems to be a critical phenomenon that depends on intimate interactions of intrinsically firing and dormant cells. When intrinsically firing cells reach a critical number, they prevail: they not only fire AP themself, but also recruit a fraction of excitable dormant cells to fire APs. And, vice versa, as the sub-population of firing cells decreases, these cells become surrounded by a critical number of dormant cells, and these dormant cells suppress the entire system automaticity (for example, in scenarios 4 and 7). At the criticality, the transition from the firing state to non-firing state in SAN tissue is extremely abrupt, like a phase transition in statistical physics (Figure 8B). For example, in scenarios 4, 5b, and 7, the AP generation suddenly ceased at about 3.5, 6, and 4.5 s after simulation onset, respectively, when the system reached a criticality, and the suppressing effect of non-firing cells prevailed (see Supplementary Movies S4, 5B, 7 and Figure 8B, red circle).

The presence of non-excitable cells accounts for a substantial fraction of non-firing cells in tissue in scenarios 1 to 3 featuring substantial *g*_*CaL*_ spread (Table 1). In contrast to scenarios with *g*_*CaL*_ distributions, all cells in scenarios with *P*_*up*_ distributions and fixed *g*_*CaL*_ were excitable (albeit variously, depending on *g*_*CaL*_). This makes possible for the system to evolve to the state when all cells fire APs and, as a result, scenarios with *P*_*up*_ distributions (4,5, 6, 8, 12, 16, 18) tended to exhibit all-or-none firing (Table 1). An interesting result was that, despite the lack of cells with intrinsically chaotic firing, in many different scenarios some cells self-organized into local communities of desynchronized firing, including chaotical firing APs and low amplitude sub-threshold oscillations (Figure 4, Supplementary Figures S1–S3). This result indicates that complex patterns of AP firing (similar to that observed in SAN tissue, Figure 4 in Bychkov et al., 2020) can emerge as a new system property when cells interact within the tissue.

Because we distributed cells uniformly randomly, the critical phenomenon of the emerging automaticity in our tissue model can be interpreted in terms of Poisson clumping (Aldous, 1989), a phenomenon wherein random events (in space and/or time) have a tendency to occur in clusters, clumps, or bursts. In other words, forming cell clusters with high *g*_*CaL*_ and/or *P*_*up*_ could be a natural consequence of the random distribution. When these natural clusters of intrinsically firing cells are rare and small, their automaticity is suppressed by surrounding dormant cells (which, in turn, also tend to form clusters via Poisson clumping). The emergence of local communities of chaotically firing cells (Figure 4, Supplementary Figures S1–3), can also be attributed Poisson clumping.

A recent study (Fenske et al., 2020) showed that a mutual interaction process between firing and nonfiring cells can slow down the overall rhythm of the SAN, but the percentage of firing cells can be increased by cAMP-dependent regulation and thereby oppose enhanced responses to vagal activity. Our results are in accord with this report and provide further insights into this new phenomenon. Indeed, it is well-known that cAMP signaling enhances both *I*_*CaL*_ and SERCA pumping (i.e. *g*_*CaL*_ and *P*_*up*_ in our study). Thus, the critical nature of interactions revealed in the present study could be interpreted to indicate that increased cAMP-dependent regulation of SAN function shifts the system operation away from the critical border, deeper towards rhythmic firing area characterized by a strong recruitment of dormant cells (Scenarios 15–18, Table 1). And vice-versa, a perturbation that increases the fraction of dormant cells would slow down the system automaticity and brings it closer to the critical border.

### Cell Clustering Is yet Another Mechanism to Enhance Robustness of SAN Function

While forming functional clusters via Poisson clumping in our model is a matter of chance (albeit predictable via statistical methods (Aldous, 1989)), the real SAN tissue, as any biological tissue, can direct cell locations to optimize its function. Indeed, high resolution imaging studies in intact SAN detected clusters of cells with various types of functional activity (Figure 4 in Bychkov et al., 2020). Based on this logic, we rescued SAN tissue function in the “hopeless” scenarios 4 and 7 (Supplementary Movies S4, 7) just by reorganizing the cell locations within the tissue models (scenarios 9 and 10, Supplementary Movies S9, 10). Dormant cells were “kicked” to SAN periphery, while intrinsically firing cells (clustered in the grid center) supported AP firing of each other. As a result, the tissue automaticity was not only rescued, but a substantial fraction of dormant cells (at the border of the firing/non-firing cells) was recruited to fire APs, as evident from the increase of the percentage of firing cells (scenarios 9 and 10 in Table 1). Thus, cell clustering (e.g. with respect to *g*_*CaL*_, *P*_*up*_, and perhaps other parameters) may represent an additional mechanism by which SAN tissue can enhance its robust function at the edge of stability. A large number of local oscillators detected in intact SAN (Bychkov et al., 2020) may reflect cell clustering due to heterogeneity in *I*_*CaL*_ (yielding non-excitable cells, besides excitable cells, as described above, see also Figure 1A), but the fact that the cell clusters operate at different frequencies point to (among perhaps other interpretations) cell heterogeneity in SR Ca pumping determined by various levels of phospholamban phosphorylation among cells or cell clusters (mimicked by *P*_*up*_ heterogeneity in our model). Indeed, heterogeneity in *P*_*up*_ created numerous oscillators operating at various frequencies in our tissue models (wide histograms in Figures 5B, 6B).

An apparent downside of cell clustering, however, is that the isolated clusters of automaticity lack physiological importance until their signals overcome the non-firing cell “barriers” to interact with other clusters and/or the rest of the SAN to ultimately deliver synchronized, rhythmic (but not metronomic) APs to atria. While this appears to be a problem in our simple 2-dimensional model of SAN tissue, the real SAN tissue is a 3-dimensional, multi-layer meshwork of long and highly branched, intertwined cells (Bychkov et al., 2020). Such tight meshwork of HCN4^+^/Conexin43^-^ cells is structured for signal transfer within and among the functional clusters, regardless of the specific nature of cell interactions that remains unknown.

### Cell Heterogeneity Is an Antiarrhythmic Mechanism

Another important result of our study is that heterogeneity in *g*_*CaL*_ or *P*_*up*_ has antiarrhythmic effect. Indeed, while the tissue models (scenarios 3 and 6) with identical cells generated chaotic firing, allowing *g*_*CaL*_ or *P*_*up*_ values to spread (but keeping same averages over the cellular network) shifted the system towards normal, rhythmic operation (Figure 3D, Supplementary Movie S3 and Figure 5C, Supplementary Movie S6). This result is remarkable and counterintuitive: when the noise of an intrinsically chaotic oscillatory system is combined with the noise of random distribution of its key parameter (*g*_*CaL*_ or *P*_*up*_) within a SAN network, it is hard to expect that such “double-noisy” system would generate normal rhythmic automaticity; but it does. However, it is important to note that such an antiarrhythmic mechanism and related potential improvement of heart function may be specific to the SAN. In cardiac muscle, excessive cell heterogeneity contributes to the induction of reentrant arrhythmia. In the case of triggered activity, the robustness by heterogeneity presented in this study may cause a robust ectopic firing. In either case, abnormal impulse initiation in atria or ventricles is proarrhythmic (rather than antiarrhythmic) and can be life-threatening.

### Weak Cell Coupling Is Required for Robust Operation of the SAN Tissue

Our simulations with different coupling strength show that weak cell coupling is required for the robust operation in our heterogeneous SAN tissue model operating at the edge of stability. Our SAN model was set as a community of loosely coupled cells with very high resistivity of cell-to-cell contacts of *ρ* = 10^4^ MΩ·m (Campana, 2015), and in many scenarios, we were able to revive SAN function in the non-firing zone by adding heterogeneity to *g*_*CaL*_ or *P*_*up*_ (Table 1). When we increased cell coupling to an intermediate setting with *ρ* = 10^3^ MΩ·m, the system notably increased the average AP cycle length (scenarios 11 and 12, Table 1) and the velocity of an apparent AP propagation (Supplementary Movie S11 vs. Supplementary Movie S2, Supplementary Movie S12 vs. Supplementary Movie S5); but when the coupling was increased further to *ρ* = 1 MΩ·m, i.e. whole tissue became almost instantly synchronized, SAN ceased operation (scenarios 13 and 14, Supplementary Movies S13, 14). This result may explain, in part, why cells are weakly connected (no CX43 was found (Boyett et al., 2006)) in the central part of the SAN, where the cardiac impulse emerges: the system of loosely coupled cells is more robust, when it operates at the edge of stability. The mechanism of this phenomenon could be linked to the same critical behavior of the system discussed above, i.e. the critical interactions of dormant and intrinsically firing cells. Indeed, dormant cells represent >50% of the cell populations in our scenarios 1–14 (Table 1), and their effect to halt SAN automaticity becomes prevailing at stronger coupling. In other terms, in loosely connected SAN tissue the intrinsically firing cells can operate when they represent a minority (and even recruit to fire dormant cells), whereas in strongly connected SAN tissue their individual behaviors are suppressed by dominating dormant cells, and the dormant cells halt the system function. Our simulations also showed that at the intermediate cell coupling, i.e. in the transition from AP firing at weak coupling towards SAN failure at strong coupling, the tissue AP firing was well-organized into cell-wide, fast-propagating waves (Supplementary Movies S11, 12). Interestingly, while such almost metronomic AP firing of SAN tissue may appear as an almost perfect operation, it actually heralds SAN failure at stronger cell coupling (Supplementary Movies S13, 14).

### Study Limitations and Future Studies

Our simulations showed that system transition from firing to non-firing state seems to be a critical phenomenon, i.e. either firing cells prevail and recruit dormant cells to fire or dormant cells prevail and halt automaticity. The question then arises: what is the minimal percentage of firing cells that is enough for SAN function? In theory, the exact border between SAN life and death in our model could be found via a parametric sensitivity analysis. However, tissue modeling is associated with high computational demand and, therefore, detailed sampling of parametric space, like we did for single cell models (Figure 3A), is infeasible. Our simulations were limited to 1) 7.5 s or 25 s of tissue function; 2) two specific key parameters of the cell coupled clock-system, *g*_*CaL*_ and *P*_*up*_; 3) a simple model of SAN tissue of a square grid of 25 × 25 cells; 4) only 1 cell size in the tissue (i.e. same cell electric capacitance); 5) only 1 cell model type (Maltsev and Lakatta, 2009); 6) only 3 cases of cell coupling strength; 7) model scenarios close to the bifurcation line; 8) only two types of cell distribution: uniformly random and spiral clustering.

Although entire parametric space of *P*_*up*_ - *g*_*CaL*_ have not been examined, our results show that the SAN can operate with the percentage of intrinsically firing cells as low as 40%. Based on our data in Table 1, we constructed a summary graph showing the margin between “firing” and “non-firing” SAN for tissue simulations scenarios with uniformly random distribution of *P*_*up*_ and *g*_*CaL*_ (Figure 8B). Orange line shows a hypothetical phase transition between the two states. This is, of course, a rough estimate, formally based on simulations presented here with all aforementioned limitations.

To precisely answer the question of the SAN life-or-death border, however, is difficult. The SAN may engage protective mechanisms to avoid failure, such as complex neuronal control (Hanna et al., 2021), areas that are unresponsive to acetylcholine (Opthof, 2007), complex morphological structures including cell clustering (discussed above), etc. For example, while SAN lacks automaticity with 30% of functional cells in scenario 7, its function can be rescued by rearranging (clustering) the same cells within the grid in scenario 9 (Figure 6). Thus, the exact border of the critical interactions between firing and dormant cells was not fully characterized in the present study and merits further, more detailed numerical and analytical studies, including bifurcation analysis (Kurata et al., 2012) and statistical physics approaches to describe phase transition-like behaviors in heart cells and tissues (Maltsev et al., 2017a; Ashikaga and Asgari-Targhi, 2018). One specific theoretical approach to describe such behaviors could be via Ising model, because firing cells favor firing states of their neighbors, whereas silent cells favor silent sates of their neighbors, like opposite spin orientations in ferromagnetism (Onsager, 1944).

Future theoretical studies can also explore contributions of other ion currents such as *I*_*Na*_ (Lei et al., 2004), *I*_*CaT*_ (Ono and Iijima, 2005; Tanaka et al., 2008), and funny current (Honjo et al., 1996; Monfredi et al., 2017), phosphorylation of Ca cycling proteins (phospholamban and RyRs), autonomic modulation, cell network structure, including cell connectivity and interactions in three dimensions, interactions with other cell types (e.g. fibroblasts (Camelliti et al., 2004) and telocytes (Mitrofanova et al., 2018)), mechano-sensitivity (Quinn and Kohl, 2012), endogenous Ca buffers, pathological conditions, aging, etc. Another important aspect for future studies is a very high collagen content in SAN compared to other regions of the heart, and this is thought to have important impacts on how many SAN cells couple to each other and in what pattern (MacDonald et al., 2020). SAN cells exhibit diverse morphology, including elongated spindle shape cells and spider shape cells with varying number of irregularly shaped branches (Verheijck et al., 1998). How the number of connected neighboring cells alter the system behavior remains to be determined.

## Conclusion

Natural heterogeneity of pacemaker cells increases robustness of cardiac pacemaker function, i.e. the SAN tissue populated by heterogeneous cells can generate normal automaticity within the cell parameter ranges, whereas SAN populated by identical cells would exhibit either dysrhythmic AP firing or complete lack of automaticity. This effect to enhance pacemaker function at the edge of the system stability is synergistic with respect to heterogeneity of Ca and membrane clocks, and it is not due to a simple summation of activity of intrinsically firing cells that are naturally present in heterogeneous SAN. These firing cells critically interact with intrinsically non-firing cells (dormant cells) and either recruit many of them to fire, granting SAN automaticity, or non-firing cells suppress firing cells and abruptly halt SAN tissue automaticity (Figure 8). Clustering cells with specific cell parameters provides an additional mechanism to enhance robustness of SAN function. Robust operation of heterogeneous SAN tissue requires weak cell coupling, a known property of the central area of SAN where cardiac impulse emerges; stronger cell coupling reduces AP firing rate and ultimately halts SAN automaticity at the edge of stability. Our results provide new insights for SAN operation that can be helpful to understanding normal SAN function especially at low rates (near criticality) and in HCN4^+^/Conexin43^-^ cell communities (Bychkov et al., 2020; Fenske et al., 2020), as well as with respect to SAN function in normal aging (linked to deficient cAMP-PKA-Ca signaling (Liu et al., 2014)) and pathological conditions, such as sick sinus syndrome and SAN arrhythmias (Dobrzynski et al., 2007; John and Kumar, 2016).

## Data Availability

The raw data supporting the conclusions of this article will be made available by the authors, without undue reservation.

## Supplementary Material

Supplementary Material Guide It includes supplementary computer code, supplemental figures S1, S2, and S3 and 22 supplemental movies. Each movie illustrates the result of numerical simulation of SAN tissue (25x25 cells) lasted 7.5 s in the respective model scenario described in Table 1. V_m_ dynamics in each cell in the grid was color-coded via red shade from −60 mV (pure black) to 20 mV (pure red). Simulation time is displayed in the upper left corner. The movies were distributed in 10 supplementary Power Point files as follows: Presentation 1.PPTX: Movie 1. Presentation 2.PPTX: Movie 2a, Movie 2b, Movie 2c. Presentation 3.PPTX: Movie 3. Presentation 4.PPTX: Movie 4. Presentation 5.PPTX: Movie 5a, Movie 5b, Movie 5c. Presentation 6.PPTX: Movie 6. Presentation 7.PPTX: Movie 7, Movie 8. Presentation 8.PPTX: Movie 9, Movie 10. Presentation 9.PPTX: Movie 11, Movie 12, Movie 13, Movie 14. Presentation 10.PPTX: Movie 15, Movie 16, Movie 17, Movie 18. Specific model parameters in each movie are given in respective movie legends in the Power Point files.

## Abbreviations

AP, action potential; CaMKII, Ca/calmodulin-dependent protein kinase II; CUDA, Compute Unified Device Architecture; I_CaL_, L-type Ca current; g_CaL_, maximum conductance of L-type Ca current; SR, sarcoplasmic reticulum; RyR, ryanodine receptor (Ca release channel); SAN, sinoatrial node; SERCA2a, SR Ca pump; P_*up*_, maximum rate of SR Ca pumping by SERCA2a; PKA, protein kinase A; GPU, graphics processing unit; V_m_, membrane potential.
