# Supplementary figures and images for: Functional Heterogeneity of Cell Populations Increases Robustness of Pacemaker Function in a Numerical Model of the Sinoatrial Node Tissue
Source: Front Physiol. 2022 Apr 27;13:845634. doi: 10.3389/fphys.2022.845634 (PMC9091312; doi:10.3389/fphys.2022.845634)

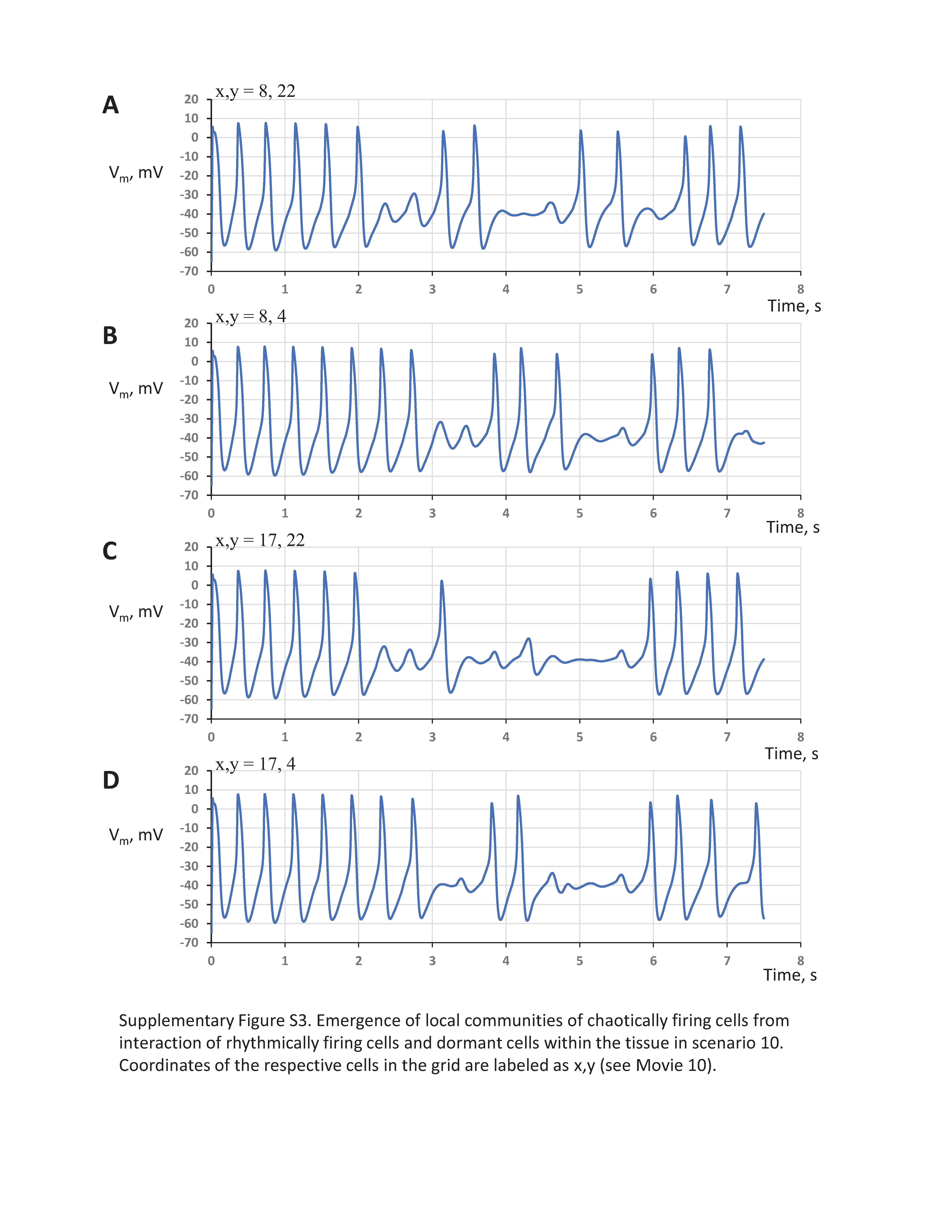

Supplement: Supplementary file 1 [file Image3.TIFF]

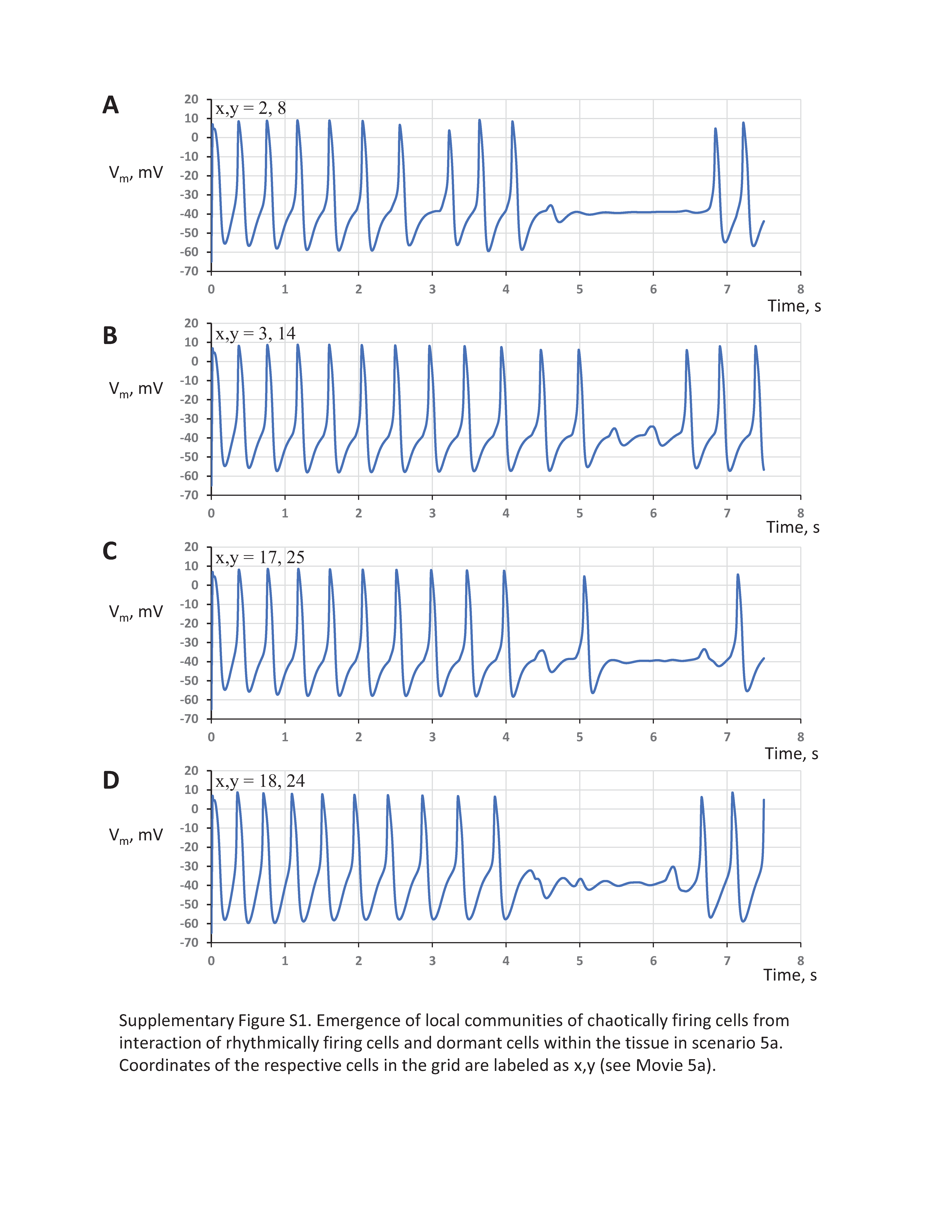

Supplement: Supplementary file 2 [file Image1.TIFF]

## Slide 1
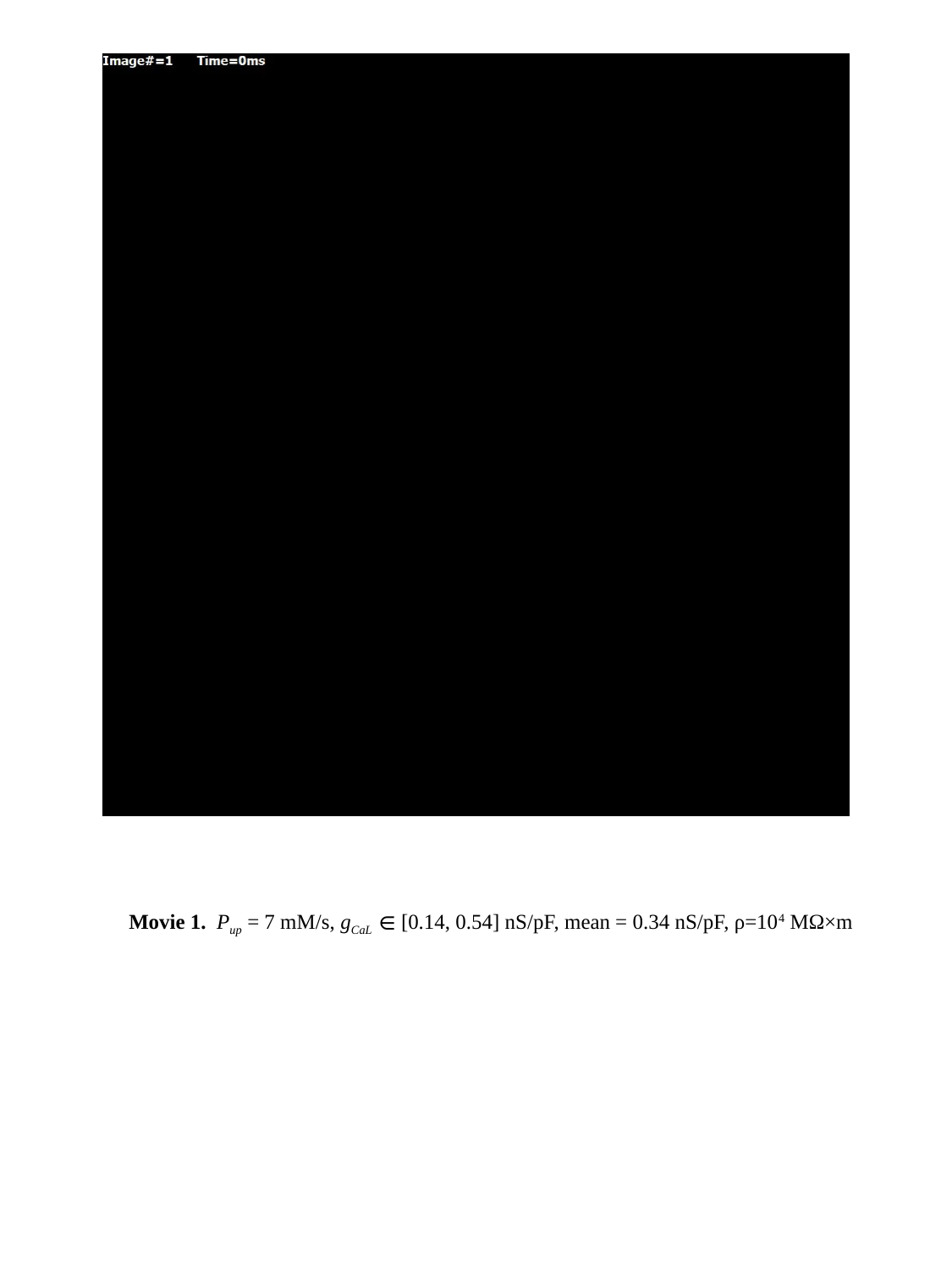

Movie 1. Pup = 7 mM/s, gCaL ∈ [0.14, 0.54] nS/pF, mean = 0.34 nS/pF, ρ=104 MΩ×m

Supplement: Supplementary file 5 [file Presentation1.PPTX]

## Slide 1
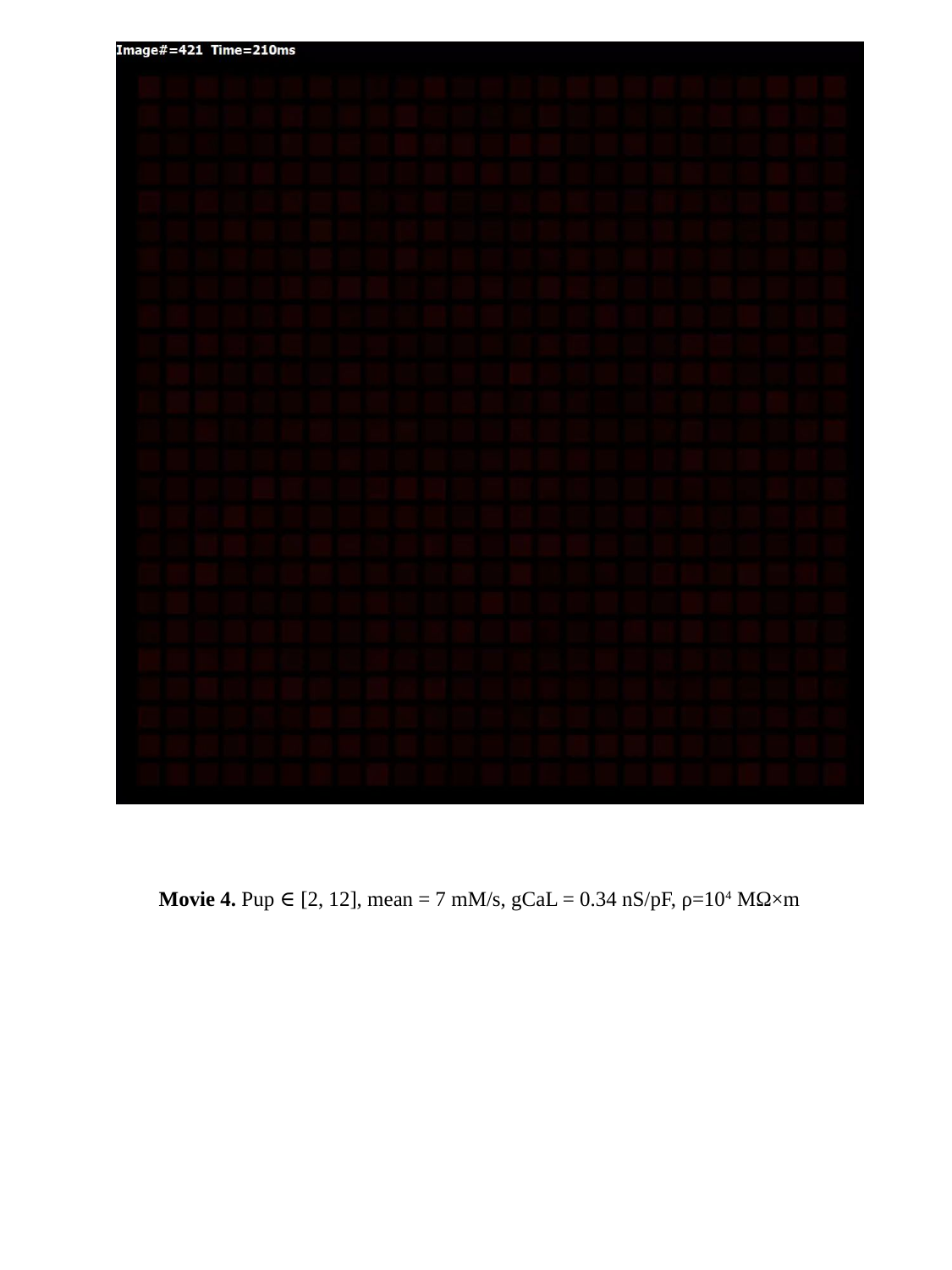

Movie 4. Pup ∈ [2, 12], mean = 7 mM/s, gCaL = 0.34 nS/pF, ρ=104 MΩ×m

Supplement: Supplementary file 6 [file Presentation4.PPTX]

## Slide 1
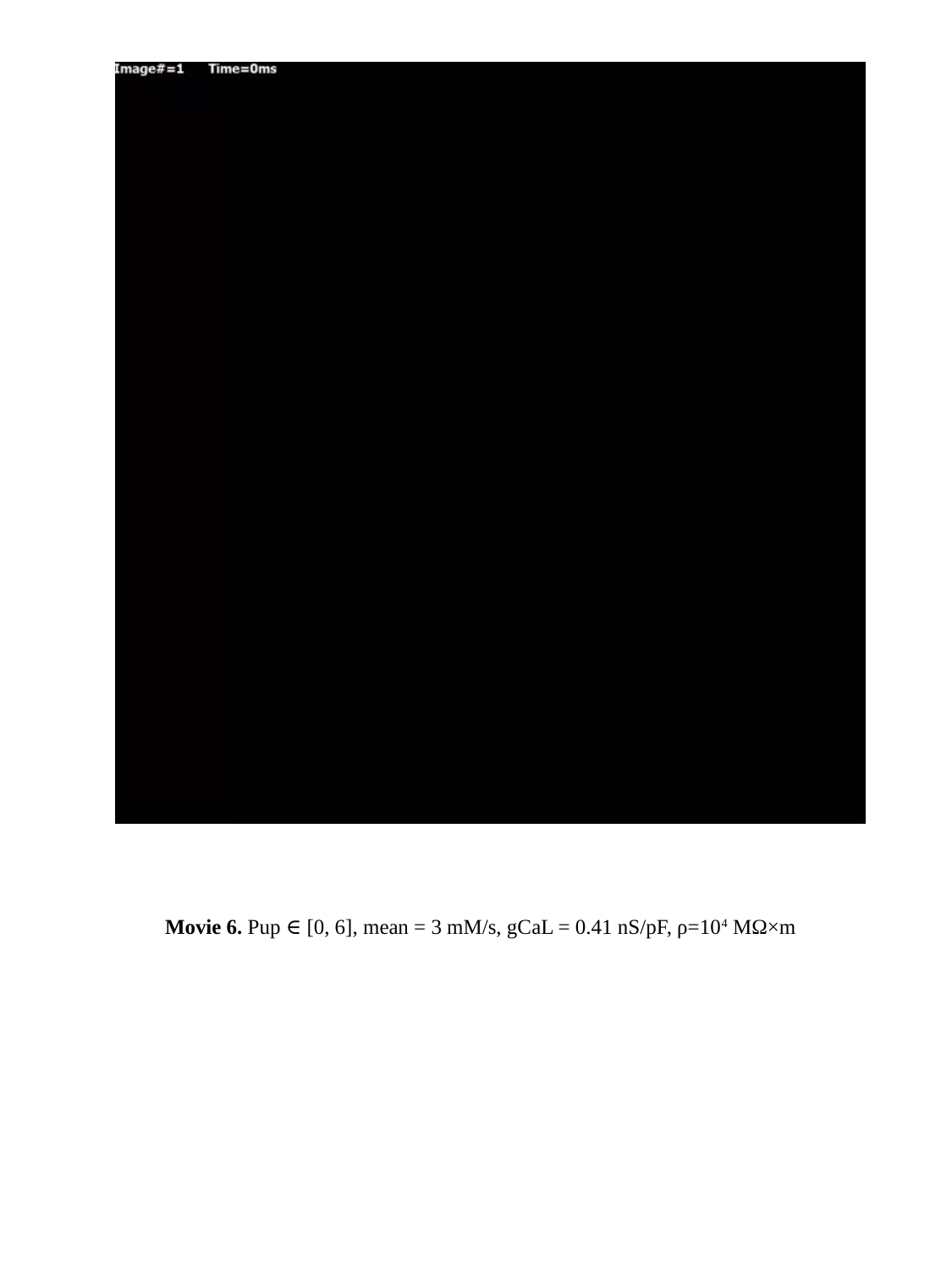

Movie 6. Pup ∈ [0, 6], mean = 3 mM/s, gCaL = 0.41 nS/pF, ρ=104 MΩ×m

Supplement: Supplementary file 7 [file Presentation6.PPTX]

## Slide 1
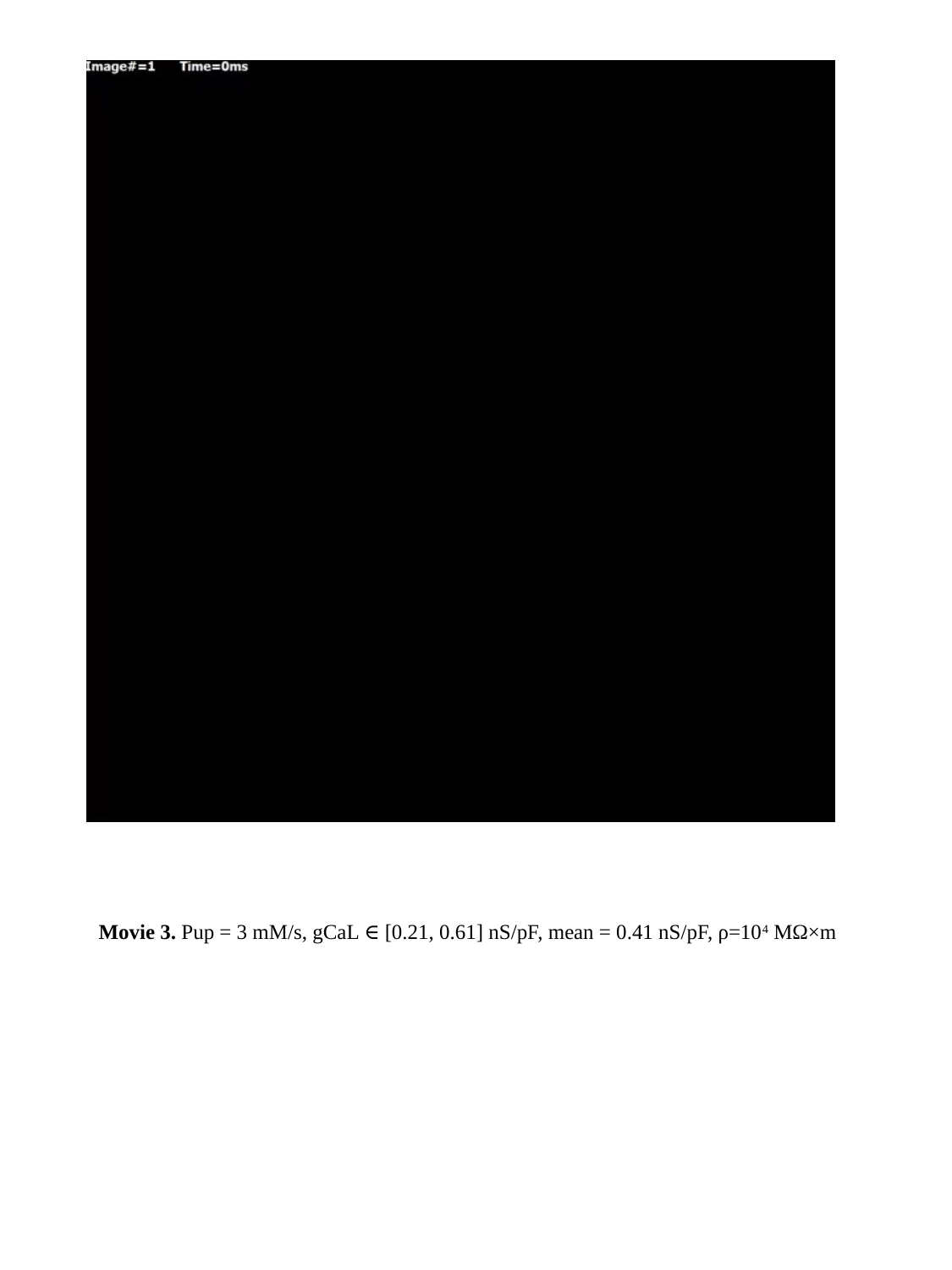

Movie 3. Pup = 3 mM/s, gCaL ∈ [0.21, 0.61] nS/pF, mean = 0.41 nS/pF, ρ=104 MΩ×m

Supplement: Supplementary file 10 [file Presentation3.PPTX]

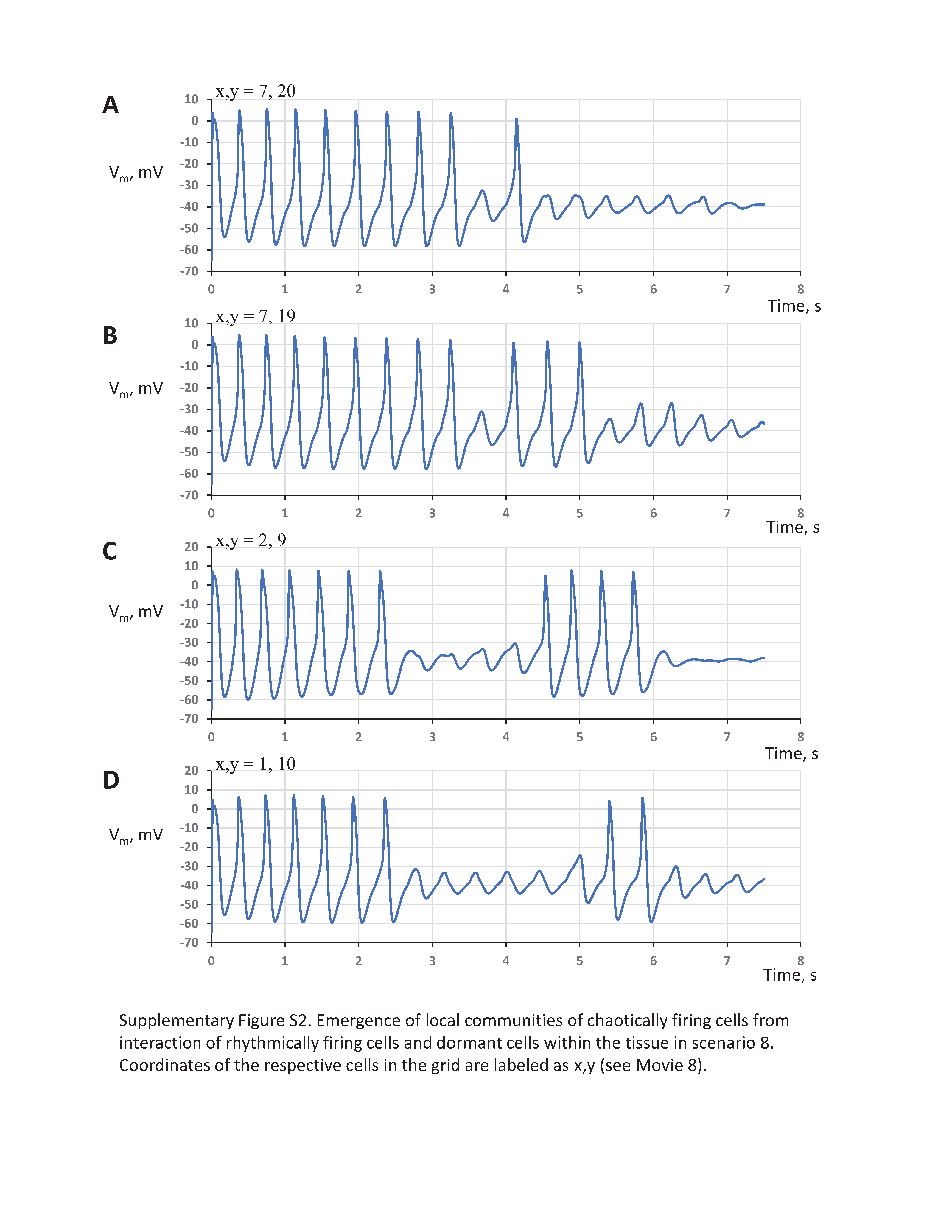

Supplement: Supplementary file 12 [file Image2.TIFF]

## Slide 1
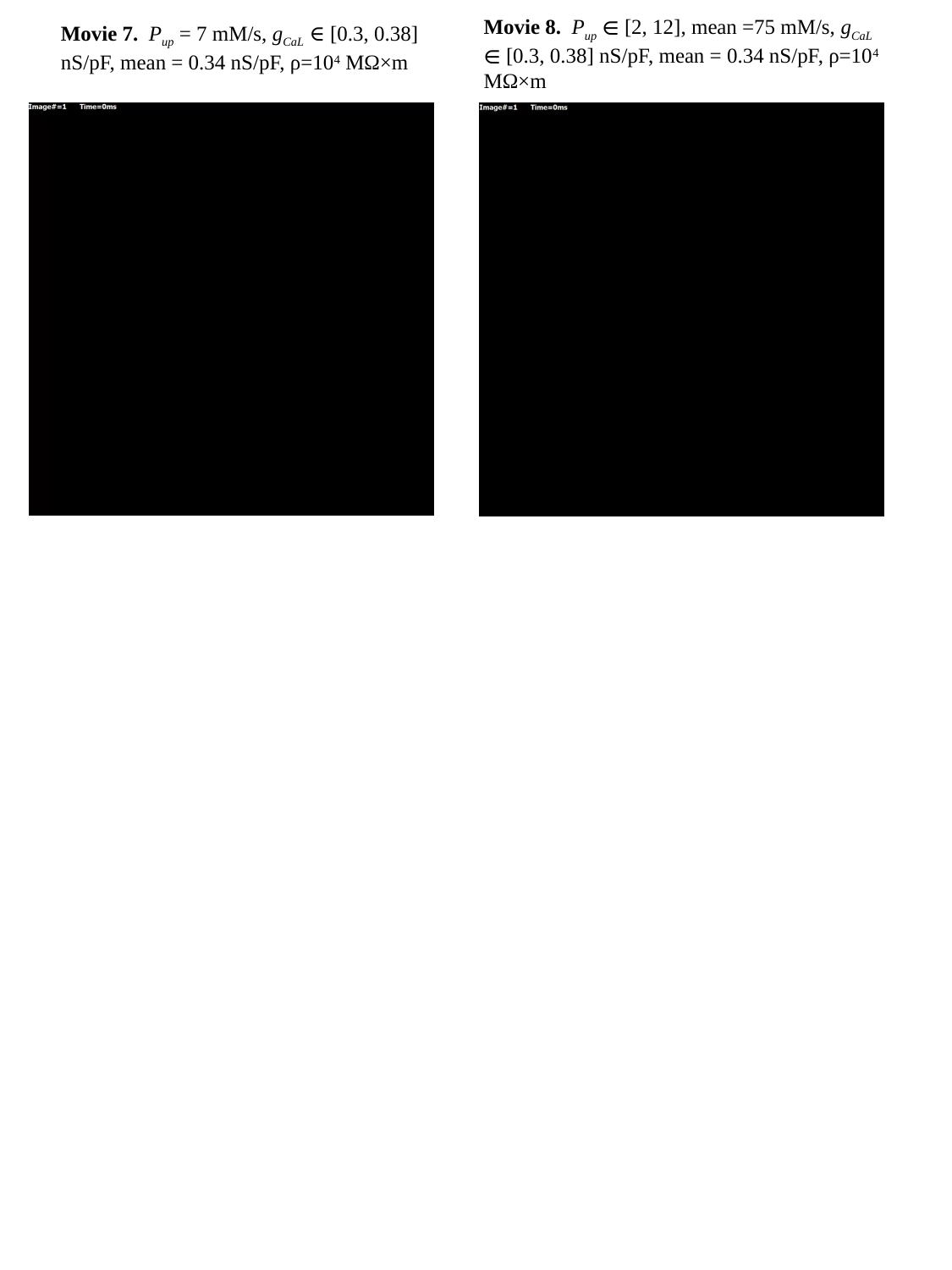

Movie 8. Pup ∈ [2, 12], mean =75 mM/s, gCaL ∈ [0.3, 0.38] nS/pF, mean = 0.34 nS/pF, ρ=104 MΩ×m
Movie 7. Pup = 7 mM/s, gCaL ∈ [0.3, 0.38] nS/pF, mean = 0.34 nS/pF, ρ=104 MΩ×m

Supplement: Supplementary file 13 [file Presentation7.PPTX]
