# Supplementary material for: Functional Heterogeneity of Cell Populations Increases Robustness of Pacemaker Function in a Numerical Model of the Sinoatrial Node Tissue
Source: Front Physiol. 2022 Apr 27;13:845634. doi: 10.3389/fphys.2022.845634 (PMC9091312; doi:10.3389/fphys.2022.845634)
Supplement: Supplementary file 3 [file Presentation9.PPTX]

## Slide 1
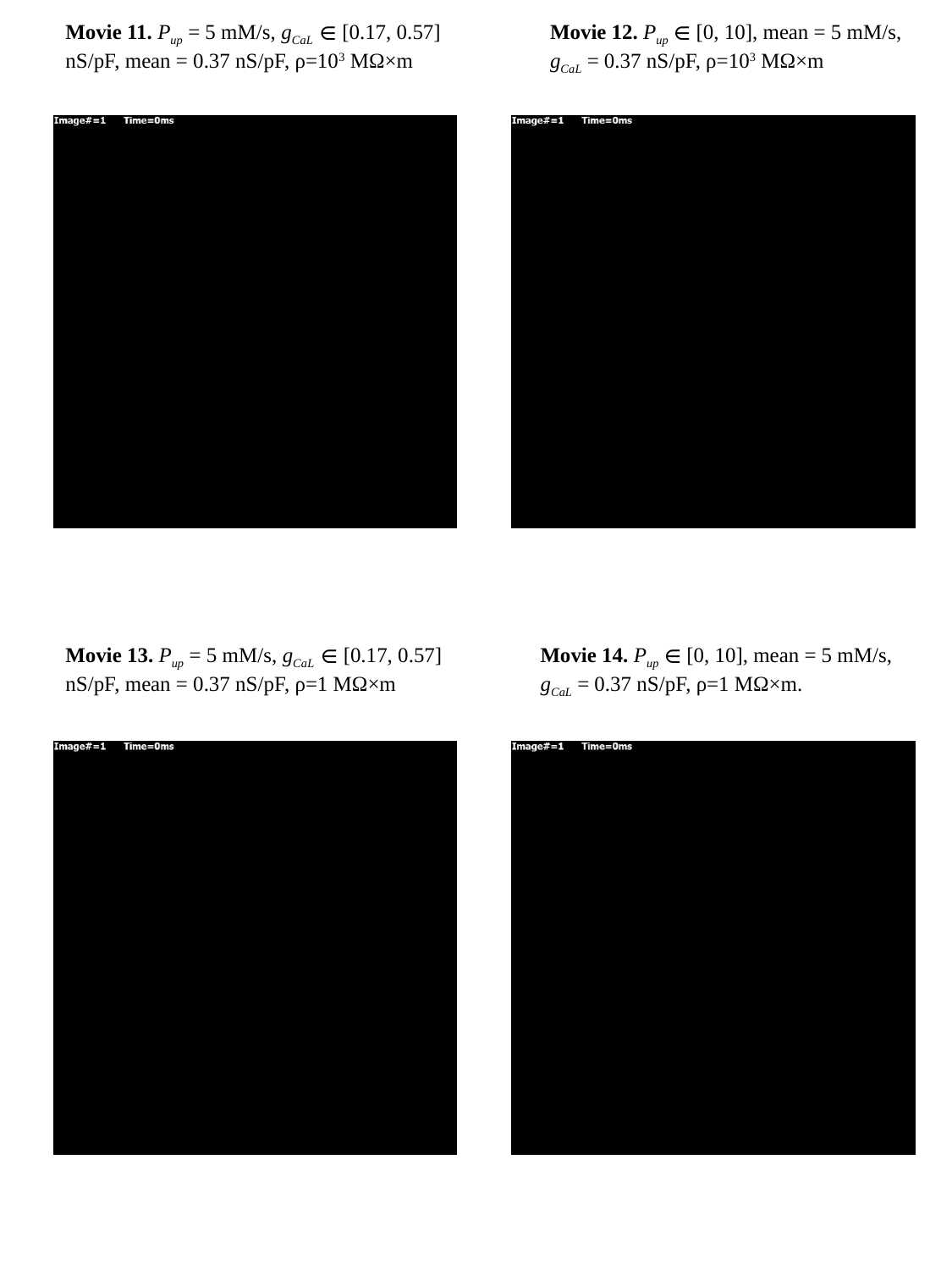

Movie 11. Pup = 5 mM/s, gCaL ∈ [0.17, 0.57] nS/pF, mean = 0.37 nS/pF, ρ=103 MΩ×m
Movie 12. Pup ∈ [0, 10], mean = 5 mM/s, gCaL = 0.37 nS/pF, ρ=103 MΩ×m
Movie 13. Pup = 5 mM/s, gCaL ∈ [0.17, 0.57] nS/pF, mean = 0.37 nS/pF, ρ=1 MΩ×m
Movie 14. Pup ∈ [0, 10], mean = 5 mM/s, gCaL = 0.37 nS/pF, ρ=1 MΩ×m.
