# Supplementary material for: Functional Heterogeneity of Cell Populations Increases Robustness of Pacemaker Function in a Numerical Model of the Sinoatrial Node Tissue
Source: Front Physiol. 2022 Apr 27;13:845634. doi: 10.3389/fphys.2022.845634 (PMC9091312; doi:10.3389/fphys.2022.845634)
Supplement: Supplementary file 4 [file Presentation10.PPTX]

## Slide 1
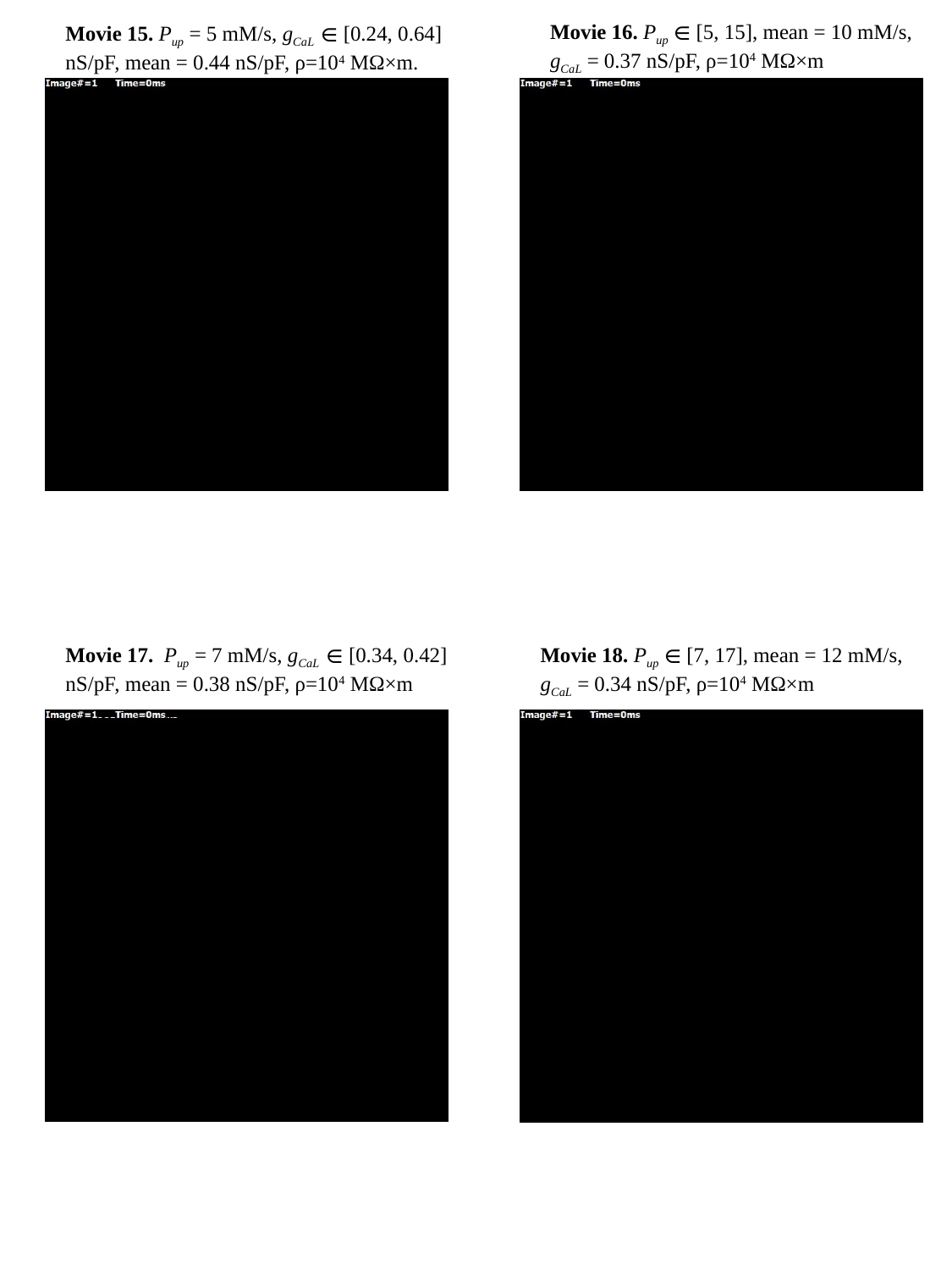

Movie 16. Pup ∈ [5, 15], mean = 10 mM/s, gCaL = 0.37 nS/pF, ρ=104 MΩ×m
Movie 15. Pup = 5 mM/s, gCaL ∈ [0.24, 0.64] nS/pF, mean = 0.44 nS/pF, ρ=104 MΩ×m.
Movie 17. Pup = 7 mM/s, gCaL ∈ [0.34, 0.42] nS/pF, mean = 0.38 nS/pF, ρ=104 MΩ×m
Movie 18. Pup ∈ [7, 17], mean = 12 mM/s, gCaL = 0.34 nS/pF, ρ=104 MΩ×m
