# Supplementary material for: Functional Heterogeneity of Cell Populations Increases Robustness of Pacemaker Function in a Numerical Model of the Sinoatrial Node Tissue
Source: Front Physiol. 2022 Apr 27;13:845634. doi: 10.3389/fphys.2022.845634 (PMC9091312; doi:10.3389/fphys.2022.845634)
Supplement: Supplementary file 8 [file DataSheet1.PDF]

Supplementary computer code.

Algorithm to create a cluster of cells (C)

```
int n = width*length; //SAN grid size
std::vector<double> vector_random_nums;
for (int i = 0; i < n; i++) {
    vector_random_nums.push_back(dev_randNums[i]); //Create copy of random numbers
}
std::sort(vector_random_nums.begin(), vector_random_nums.end(), std::greater<double>());

int x, y, dx, dy;
x = y = dx = 0;
dy = -1;
int idx_copy = 0;
int X = width;
int Y = length;
int t = std::max(X, Y);
int maxI = t * t;
for (int i = 0; i < maxI; i++) {
    if ((-X / 2 <= x) && (x <= X / 2) && (-Y / 2 <= y) && (y <= Y / 2)) {
        dev_randNums[width*(x+int(width/2)) + (y+int(length/2))] =
        vector_random_nums[idx_copy]; //Order random numbers in spiral
        idx_copy++;
    }
    if ((x == y) || ((x < 0) && (x == -y)) || ((x > 0) && (x == 1 - y))) {
        t = dx;
        dx = -dy;
        dy = t;
    }
    x += dx;
    y += dy;
}
```
