# Supplementary material for: Functional Heterogeneity of Cell Populations Increases Robustness of Pacemaker Function in a Numerical Model of the Sinoatrial Node Tissue
Source: Front Physiol. 2022 Apr 27;13:845634. doi: 10.3389/fphys.2022.845634 (PMC9091312; doi:10.3389/fphys.2022.845634)
Supplement: Supplementary file 9 [file Presentation8.PPTX]

## Slide 1
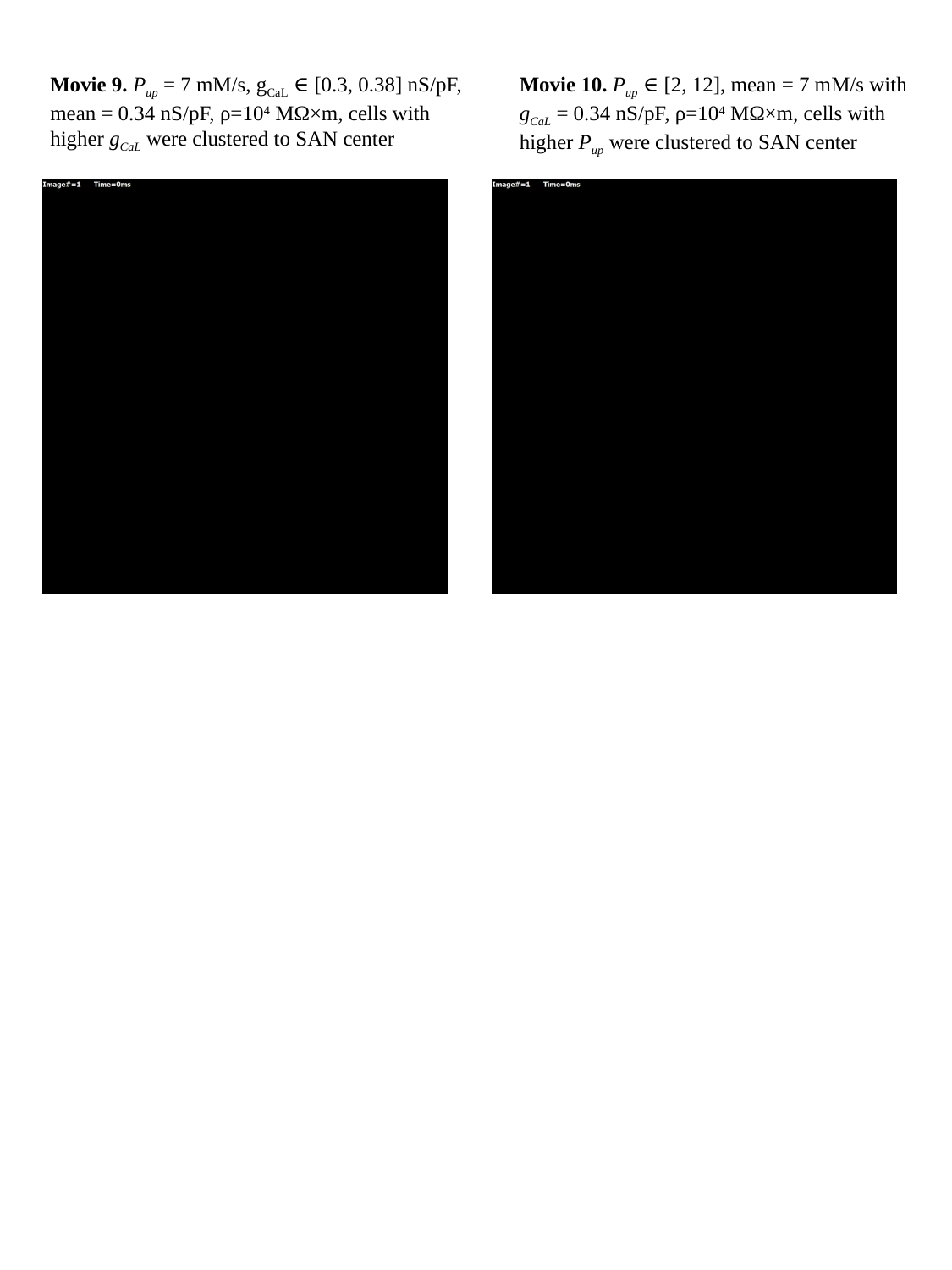

Movie 10. Pup ∈ [2, 12], mean = 7 mM/s with gCaL = 0.34 nS/pF, ρ=104 MΩ×m, cells with higher Pup were clustered to SAN center
Movie 9. Pup = 7 mM/s, gCaL ∈ [0.3, 0.38] nS/pF, mean = 0.34 nS/pF, ρ=104 MΩ×m, cells with higher gCaL were clustered to SAN center
