# Supplementary material for: Functional Heterogeneity of Cell Populations Increases Robustness of Pacemaker Function in a Numerical Model of the Sinoatrial Node Tissue
Source: Front Physiol. 2022 Apr 27;13:845634. doi: 10.3389/fphys.2022.845634 (PMC9091312; doi:10.3389/fphys.2022.845634)
Supplement: Supplementary file 11 [file Presentation2.PPTX]

## Slide 1
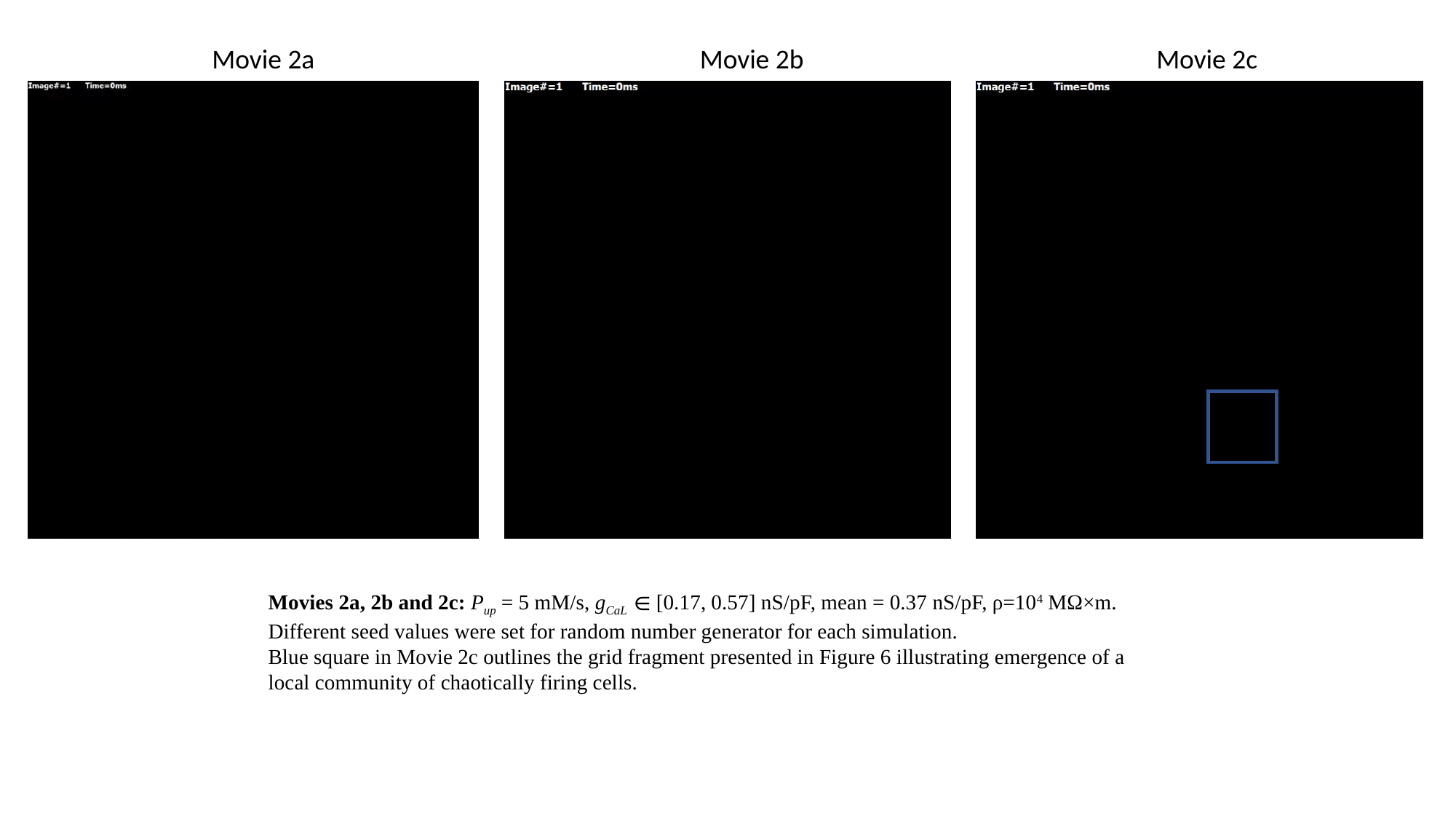

Movie 2a
Movie 2b
Movie 2c
Movies 2a, 2b and 2c: Pup = 5 mM/s, gCaL ∈ [0.17, 0.57] nS/pF, mean = 0.37 nS/pF, ρ=104 MΩ×m. Different seed values were set for random number generator for each simulation.
Blue square in Movie 2c outlines the grid fragment presented in Figure 6 illustrating emergence of a local community of chaotically firing cells.
