# Supplementary material for: Functional Heterogeneity of Cell Populations Increases Robustness of Pacemaker Function in a Numerical Model of the Sinoatrial Node Tissue
Source: Front Physiol. 2022 Apr 27;13:845634. doi: 10.3389/fphys.2022.845634 (PMC9091312; doi:10.3389/fphys.2022.845634)
Supplement: Supplementary file 14 [file Presentation5.PPTX]

## Slide 1
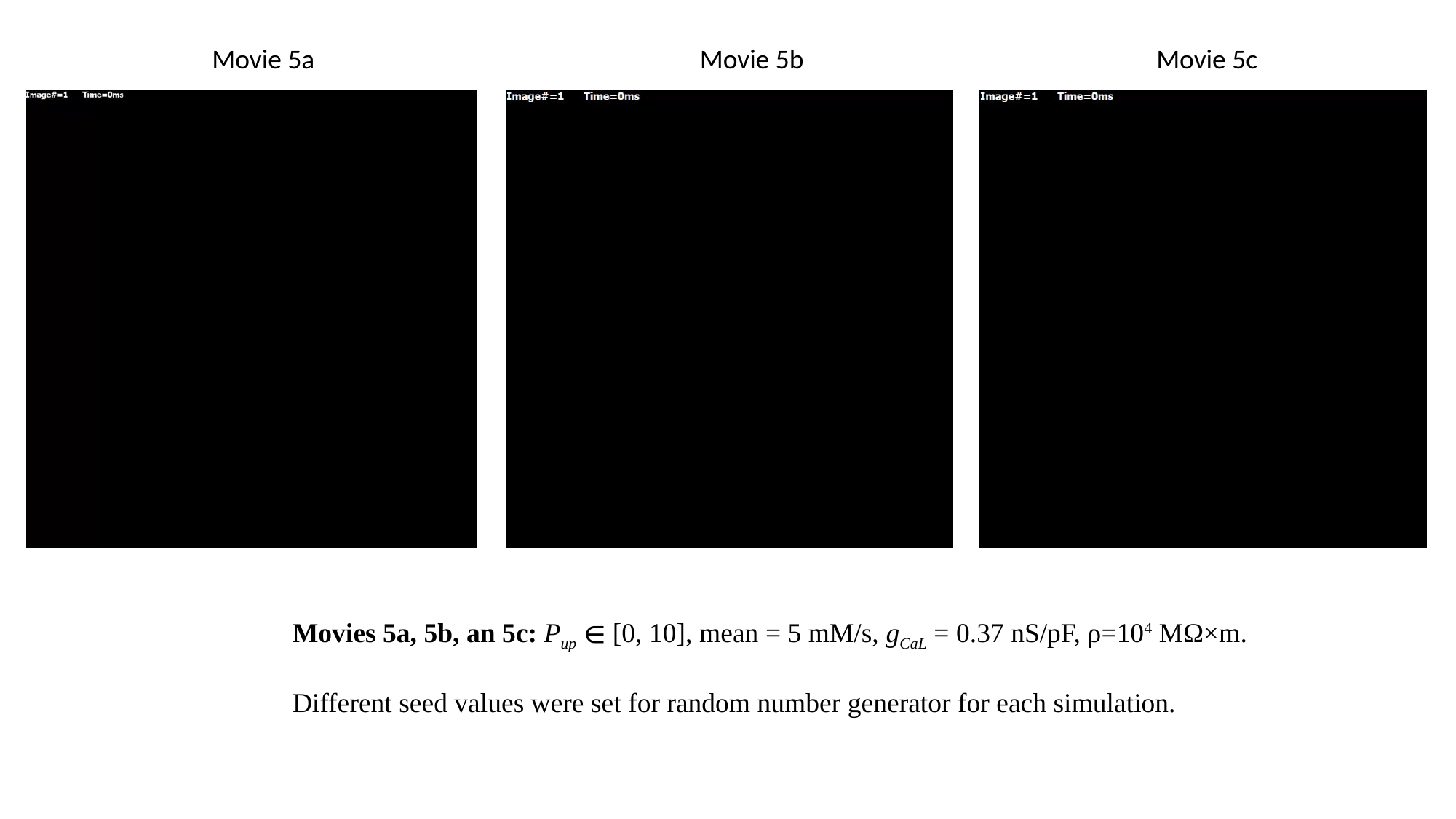

Movie 5a
Movie 5b
Movie 5c
Movies 5a, 5b, an 5c: Pup ∈ [0, 10], mean = 5 mM/s, gCaL = 0.37 nS/pF, ρ=104 MΩ×m.
Different seed values were set for random number generator for each simulation.
